# Keratoconus patients exhibit a distinct ocular surface immune cell and inflammatory profile

**DOI:** 10.1038/s41598-021-99805-9

**Published:** 2021-10-22

**Authors:** Sharon D’Souza, Archana Padmanabhan Nair, Ganesh Ram Sahu, Tanuja Vaidya, Rohit Shetty, Pooja Khamar, Ritika Mullick, Sneha Gupta, Mor M. Dickman, Rudy M. M. A. Nuijts, Rajiv R. Mohan, Arkasubhra Ghosh, Swaminathan Sethu

**Affiliations:** 1 Department of Cornea and Refractive Surgery, Narayana Nethralaya, Bangalore, India; 2 GROW Research Laboratory, Narayana Nethralaya Foundation, 3rd Floor, Narayana Nethralaya, #258/A Hosur Road, Bommasandra, Bangalore, 560099 India; 3 Manipal Academy of Higher Education, Manipal, India; 4 University Eye Clinic Maastricht, Maastricht University Medical Center, Maastricht, The Netherlands; 5 MERLN Institute for Technology-Inspired Regenerative Medicine, Maastricht University, Maastricht, The Netherlands; 6 Department of Veterinary Medicine and Surgery, University of Missouri, 1600 E. Rollins Rd, Columbia, MO 65211 USA; 7 Mason Eye Institute, School of Medicine, University of Missouri, Columbia, MO USA; 8 Harry S Truman Veterans’ Memorial Hospital, Columbia, MO USA; 9 Singapore Eye Research Institute, Singapore, Singapore

**Keywords:** Immunology, Biomarkers, Diseases, Medical research, Molecular medicine

## Abstract

Inflammatory factors have been considered to contribute to keratoconus (KC) pathogenesis. This study aims to determine the immune cells subsets and soluble inflammatory factor profile on the ocular surface of KC patients. 32 KC subjects (51 eyes) across different grades of severity and 15 healthy controls (23 eyes) were included in the study. Keratometry and pachymetry measurements were recorded. Ocular surface immune cells (collected by ocular surface wash) immunophenotyped using flow cytometry include leukocytes, neutrophils, macrophages, natural killer (NK) cells, pan-T cells, gamma delta T (γδT) cells and NKT cells. Tear fluid collected using Schirmer’s strip was used to measure 50 soluble factors by multiplex ELISA. Proportions of activated neutrophils, NK cells and γδT cells were significantly increased in KC patients. Significantly higher levels of tear fluid IL-1β, IL-6, LIF, IL-17A, TNFα, IFNα/β/γ, EPO, TGFβ1, PDGF-BB, sVCAM, sL-selectin, granzyme-B, perforin, MMP2, sFasL and IgE, along with significantly lower levels of IL-1α and IL-9 were observed in KC patients. Alterations observed in few of the immuno-inflammatory parameters correlated with grades of disease, allergy, eye rubbing and keratometry or pachymetry measurements. The observation implies a distinct immuno-inflammatory component in KC pathogenesis and its potential as an additional therapeutic target in KC management.

## Introduction

Keratoconus (KC) is characterized by focal structural changes of the cornea, including steeping and biomechanical weakening resulting in irregular astigmatism, corneal scarring, and decreased visual acuity^1,2^. Dysregulated extracellular matrix (ECM) remodeling is causally linked to various pathological conditions, including ectatic corneal diseases. Alterations in the expression of ECM core proteins (collagens, fibrin, laminin, proteoglycans) and their organization (lamellar arrangement, fibril density and diameter) are characteristic features of the KC cornea^3,4^. KC was previously presumed to be a non-inflammatory corneal ectatic disease^2^, primarily due to the lack of cardinal signs of inflammation in the cornea of KC patients. However, there is increasing evidence demonstrating the relationship between KC and aberrant levels of various inflammatory factors, including immune cells, either locally (i.e., ocular surface; cornea) or in the systemic circulation of KC patients. Furthermore, the increased risk of KC in subjects with immunological conditions, such as atopy and allergy, and better control of KC progression following the management of these conditions, imply the role of immune mediators in KC pathogenesis.

A variety of inflammatory factors, including IL-1β, TNFα, IL-6, IL-17A, IFNγ and MMP9 with an ability to influence ECM remodelling, are elevated in the tear fluid and/or corneal tissue of KC patients^5,6^. These could be contributed either by corneal structural cells or immune cells. However, the profile of immune cells on the ocular surface of KC patients remains unclear, but for a couple of reports on the presence of inflammatory cellular infiltrates in corneal tissue sections in a small subset of KC patients^7,8^. Further, it is known that a dynamic and orchestrated interaction between structural cells of tissues and immune cells contribute towards tissue homeostasis^9^. Hence, it would be pertinent to determine the profile of ocular surface immune cell subsets in KC patients to bridge a critical knowledge gap in KC pathobiology.

In the current study, we determined the levels of a variety of secreted factors (cytokines, chemokines, growth factors, soluble cell adhesion molecules, soluble receptors and enzymes) in the tear fluid, concurrently with proportions of leukocytes and various types of immune cell subsets (neutrophils subsets, macrophages, natural killer cells subsets, pan-T cells, gamma delta T cells and NKT cells) relevant in mucosal biology on the ocular surface of KC patients. Moreover, we analyzed the relationship between the cellular and secreted factors and disease severity. Importantly, we used non-invasive methods for sample collection, such as open eye ocular surface wash for immune cell proportions determination^10^ and Schirmer's strip-based tear fluid collection for secreted factor levels quantification^11^. These methods could be applied in the clinic to determine the immune-inflammatory status in KC patients, assist in disease stratification and guide targeted therapeutics.

## Methods

### Subjects and study design

The cross-sectional, observational study was approved by Narayana Nethralaya institutional ethics committee. Subject recruitment and sample collection procedures were conducted as per institutional guidelines and following the tenets of the Declaration of Helsinki. We obtained written informed consent before subject recruitment. Corneal topography and tomography were used to diagnose KC ^2,12^. Topography was acquired using Pentacam (OCULUS Optikgeräte GmbH, Wetzlar, Germany) upon the first presentation at the clinic. The Amsler–Krumeich classification, which utilizes biomicroscopy information, mean central keratometry measurement, spherical and cylindrical refraction change, and corneal thickness, was used to grade KC severity. In addition, the history of eye rubbing, as well as ocular and systemic allergy, were recorded. Exclusion criteria were contact lens use; ongoing ocular infection, severe ocular or systemic allergy, autoimmune or inflammatory conditions; history of penetrating keratoplasty, deep anterior lamellar keratoplasty, or any other recent ocular surgery including refractive surgery in the last three months; use of topical agents such as an anti-inflammatory or anti-allergic medication, and use of systemic medication known to alter immunological and inflammatory factors profile in the last 6 months. Based on the criteria mentioned above, a total of 32 KC subjects (51 eyes; age—22.3 ± 1.1 years; M/F—19/13) and 15 healthy controls (23 eyes; age—27.8 ± 0.9 years; M/F—9/6) were included in the study. The number of eyes based on grades of KC include forme fruste keratoconus—FFKC (6), grade-1 (14), grade-2 (8) and grade-3/4 (23). The grades are based on increasing degree of severity with grade 1 being the least severe and grade 4, the most severe form of the disease. A grade dependent increase in keratometry indices (K1, K2, Kmean and Kmax) and decrease in corneal pachymetry indices (central corneal thickness—CCT, thinnest corneal thickness—TCT) were confirmed in KC eyes (Supplementary Figure 1).

### Ocular surface immune cell collection

Immune cells on the ocular surface were obtained from ocular surface wash samples as described earlier^10^. Briefly, open eye ocular surface wash samples were collected by an ophthalmologist in an outpatient clinical setup. A needleless sterile syringe was used to gently rinse the study subject's ocular surface with sterile saline (room temperature). We used a sterile tube positioned close to the lateral canthus of the eye being irrigated to collect the runoff saline. Subsequently, 0.05% paraformaldehyde was used to fix the ocular surface wash samples and stored at 4 °C until further processing.

### Ocular surface immune cell phenotyping by flow cytometry

The proportions of various immune cell subsets on the ocular surface of control and KC subjects were determined by flow cytometry-based immunophenotyping using immune cell type-specific fluorochrome-conjugated antibodies as previously described^10^. Briefly, the stored ocular surface wash samples were centrifuged at 2000 rpm for 5 min at 4 °C. The cell pellet from the ocular surface wash sample was incubated with immune cell type-specific fluorochrome-conjugated antibody cocktails diluted in staining buffer (5% Fetal Bovine Serum in 1× Phosphate Buffer Saline, pH 7.4) with agitation (500 rpm) for 45 min at room temperature. Post incubation, the cells were washed and resuspended in 300 μl 1× Phosphate Buffer Saline, pH 7.4. Fluorochrome-conjugated antibodies specific for the various immune cell subtypes are as follows: CD45 APC-H7 (*clone 2D1*), pan leukocytes; CD66b AlexaFluor 647 (*clone G10F5*), Neutrophils; CD163 FITC (*clone GHI/61*), macrophages; CD56 PE-Cy7 (*clone B159*), Natural Killer cells; CD3 PE (*clone HIT3A*), pan-T cells and γδTCR PerCP-Cy5.5 (*clone B1*), gamma delta T cells. The stated fluorochrome-conjugated antibodies were procured from (BD Biosciences, USA). Data acquisition was performed on a flow cytometer (BD FACSCanto™ II cell analyzer, BD Biosciences, USA) with BD FACSDiva software (BD Biosciences, USA) and acquired data were analyzed using FCS Express 6 (De Novo Software, USA). Post-acquisition compensation was conducted using single stained controls. Further, specific cell populations were identified, and regions were assigned based on universal negative and fluorescence minus one control. The manual gating strategy followed for immune cell subsets identification is shown in Supplementary Figure 2.

### Tear fluid collection

Tear fluid samples were obtained from study subjects as previously described^11^. Briefly, sterile Schirmer's strips was used to collect the tear fluid. The collected strips were then stored in sterile microcentrifuge tubes at − 80 °C until further processing. Tear fluid proteins were eluted from the Schirmer's strip by the agitation of cut pieces of Schirmer's strip in 300 μl of sterile 1× PBS for 2 h at 4 °C followed by centrifugation. The eluted tear fluid (300 μl) was collected in a fresh sterile microcentrifuge tube was used for further downstream analyses.

### Tear fluid soluble factors measurements

The levels of cytokines (IL-1α, IL-1β, IL-2, IL-6, LIF, IL-9, IL-10, IL-12/IL23p40, IL-12p70, IL-13, IL-17A, IL-18, IL-21, TNFα, IFNα, IFNβ, IFNγ), chemokines (MCP1/CCL2, RANTES/CCL5, Eotaxin/CCL11, IL-8/CXCL8, MIG/CXCL9, IP-10/CXCL10, ITAC/CXCL11, Fractalkine/CX3CL1), growth factors (TGFβ1, bFGF, HGF, EPO, PDGF-AA, PDGF-BB, VEGF), soluble cell adhesion molecules (sICAM1, sVCAM, sL-selectin, sP-selectin), soluble receptors (sTNFRI, sTNFRII, sIL-1R1), enzymes (MMP2, MMP9, TIMP1, MPO, NGAL, Angiogenin) and other secreted proteins (Granzyme-B, Perforin, IgE, sFasL, β2microglobulin) in the tear fluid were simultaneously measured by multiplex ELISA. IL-1α, IL-1β, IL-2, IL-6, IL-9, IL-10, IL-12/IL23p40, IL-12p70, IL-13, IL-17A, IL-21, TNFα, IFNα, IFNγ, MCP1/CCL2, RANTES/CCL5, Eotaxin/CCL11, IL-8/CXCL8, MIG/CXCL9, IP-10/CXCL10, ITAC/CXCL11, Fractalkine/CX3CL1, TGFβ1, bFGF, VEGF, sICAM1, sVCAM, sL-selectin, sP-selectin, sTNFRI, sTNFRII, sIL-1R1, Angiogenin, IgE and sFasL were measured using Cytometric Bead Array (BD™ CBA Human Soluble Protein Flex Set System, BD Biosciences, USA) as per manufacturer’s instruction. Similarly, LIF, IL-18, IFNβ, HGF, EPO, PDGF-AA, PDGF-BB, MMP2, MMP9, TIMP1, MPO, NGAL, Granzyme-B, Perforin, and β2microglobulin were measured using LEGENDplex (Biolegend Inc, USA) according to manufacturer's instructions. A flow cytometer (BD FACSCanto™ II, BD Biosciences, USA) with BD FACSDiva software (BD Biosciences, USA) was used to acquire the beads and record signal intensities for both Cytometric Bead Array and LEGENDplex assays. Analyte specific standards were utilized to determine the absolute concentrations of the analytes using analytical softwares—FCAP array Version 3.0 (BD Biosciences, USA) for Cytometric Bead Array and LEGENDplex™ Data Analysis Software Suite (Biolegend Inc, USA) for LEGENDplex assays. Wetting length of Schirmer's strip during tear fluid collection and tear elution buffer volume was used to determine the dilution factor to calculate the final concentration of the analytes in the tear fluid sample of the study subjects.

### Statistical analysis

The findings are reported as Mean ± Standard error of Mean (SEM) and represented as bar graphs. Differences in the various parameters between the groups were tested for statistical significance by Mann–Whitney test using GraphPad Prism 8.0 (GraphPad Software, Inc., La Jolla, CA, USA). Correlation among the various study parameters was determined by Spearman Rank correlation tests using MedCalc^®^ Version 12.5 (MedCalc Software, Ostend, Belgium). P < 0.05 was considered to be statistically significant.

## Results

### Ocular surface immune cell subsets proportions in patients with KC

**Figure 1:**
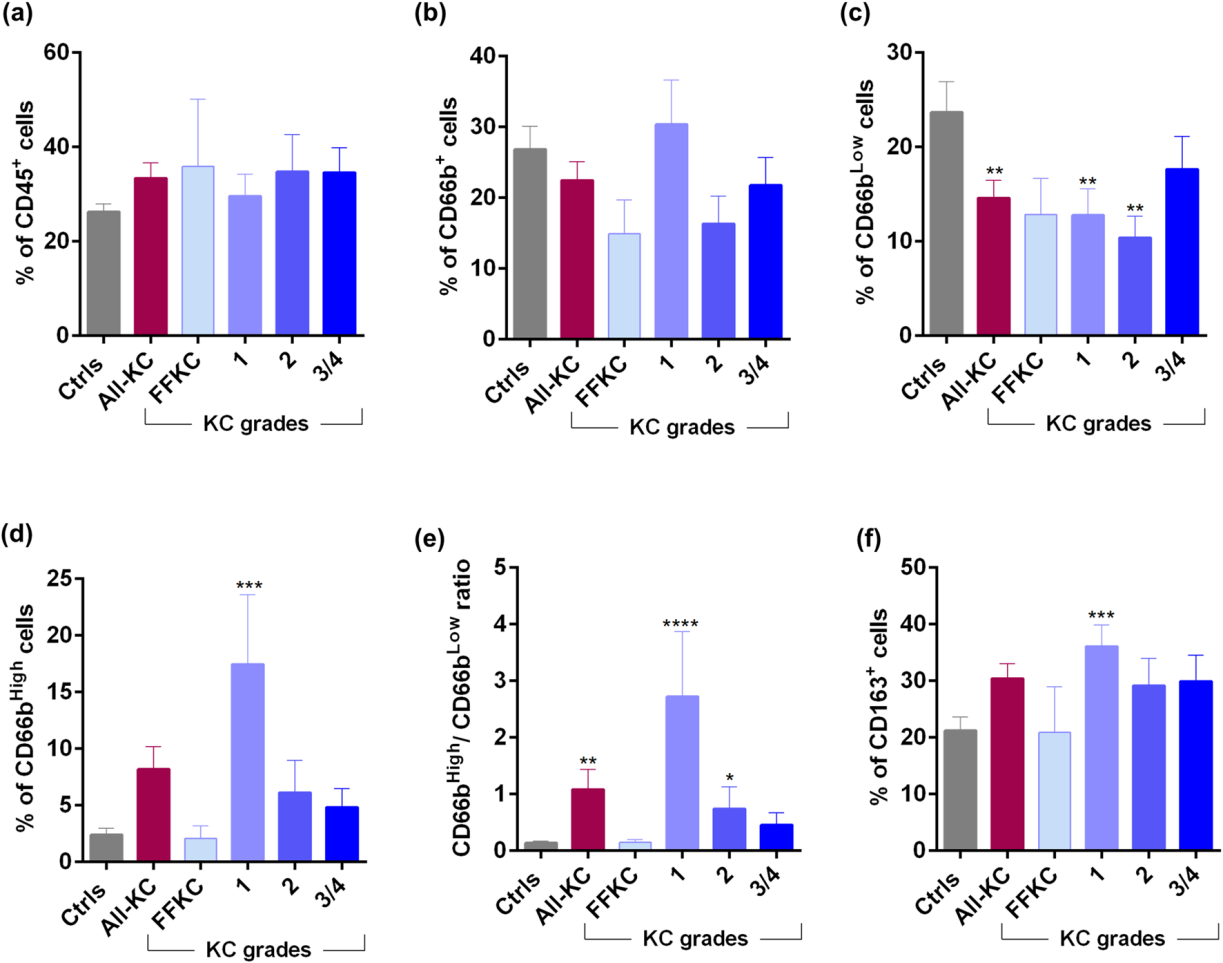
Ocular surface leukocytes, neutrophil subsets and macrophage proportions in KC patients: (**a**) Graph indicates the percentage of CD45^+^ (Pan leukocyte marker) cells in ocular surface wash samples of eyes with different grades of KC. (**b**–**e**) Graphs indicate the percentage of CD45^+^CD66b^Total^ (Neutrophils), CD45^+^CD66b^Low^ (inactive neutrophils), CD45^+^CD66b^High^ (activated neutrophils) and the ratio of activated to inactive neutrophils (CD66b^High^/CD66b^Low^) within the leukocyte population in ocular surface wash samples of eyes with different grades of KC. (**f**) Graph indicates percentages of CD45^+^CD163^+^ (macrophages) cells within the leukocyte population in ocular surface wash samples from subjects of eyes with different grades of KC. Ctrls—Controls; FFKC—Forme fruste keratoconus; All-KC—all the grades of keratoconus combined; SEM—standard error of the mean; Controls (23 eyes), All-KC (51 eyes), FFKC (6 eyes), grade 1 KC (14 eyes), grade 2 KC (8 eyes), grade 3 or 4 (23 eyes); Bar graphs represent Mean ± SEM; *P < 0.05, **P < 0.01,***P < 0.001, ****P < 0.0001, Mann–Whitney test.

The proportion of leukocytes (CD45^+^) was not significantly different between controls and various grades of KC (Fig. 1a). A decrease in the proportion of total neutrophils (CD45^+^CD66b^+^), though not statistically significant, was observed in KC compared to controls (Fig. 1b). However, a significant decrease in the proportion of inactive neutrophils (CD45^+^CD66b^Low^) along with a significant increase in the proportions of activated neutrophils (CD45^+^CD66b^High^) was observed in KC compared to controls (Fig. 1c–e). More specifically, grade-1 and -2 KC was noted to be contributing to this observation (Fig. 1c–e). A significant increase in the proportion of macrophages (CD45^+^CD163^+^) was observed in grade-1 KC compared to controls (Fig. 1f).

**Figure 2:**
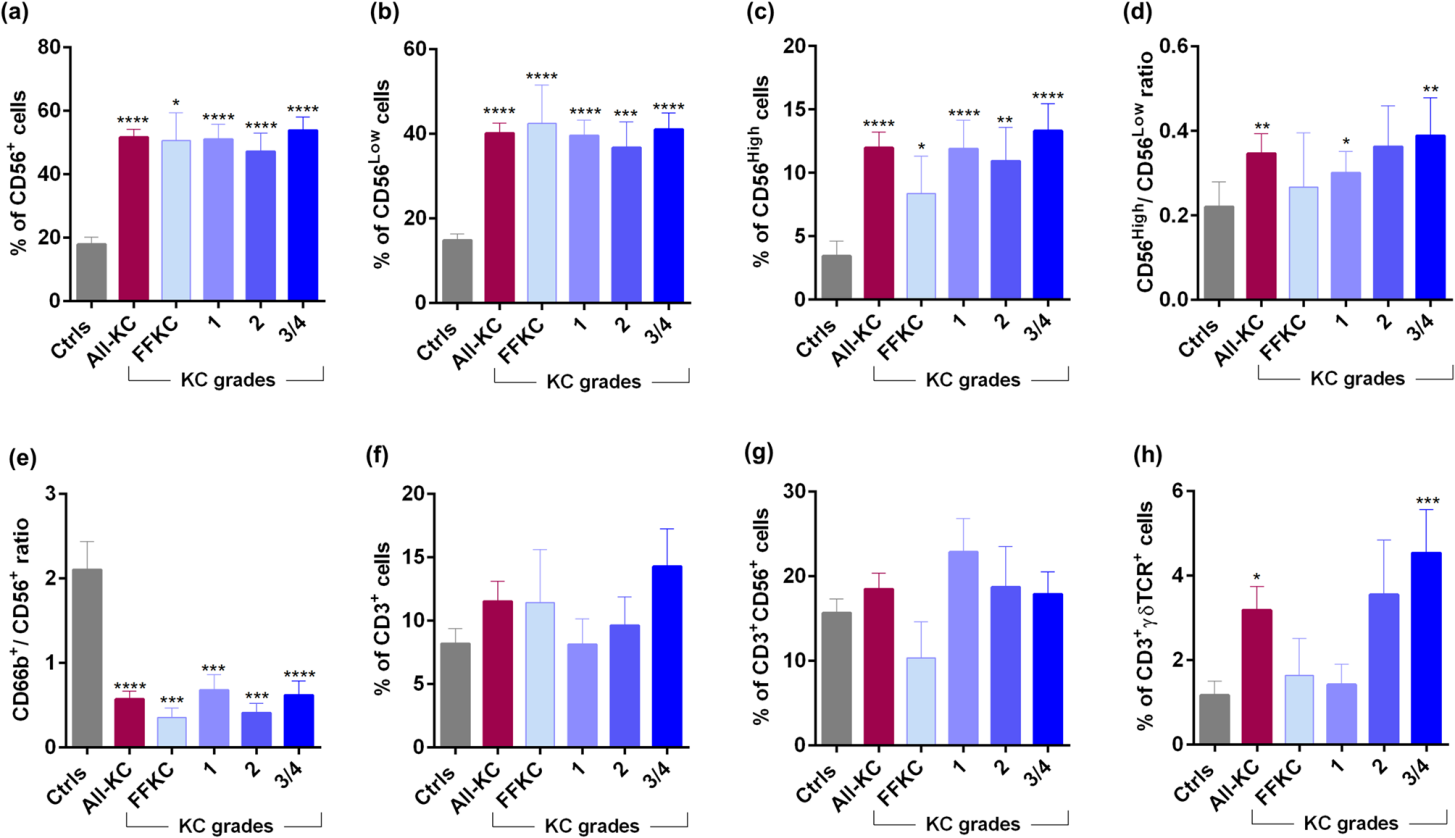
Ocular surface natural killer cells subsets and T cell subsets proportions in KC patients: The graphs indicate the percentage of CD45^+^CD56^Total^ (Total Natural Killer—NK) cells (**a**), CD45^+^CD56^Low^—cytotoxic NK cells (**b**) and CD45^+^CD56^High^—cytokine producing low cytotoxic NK cells (**c**) within the leukocyte population in ocular surface wash samples of eyes with different grades of KC. The graph in panel (**d**) represent the ratio of cytotoxic NK cells to cytokine producing NK cells—CD56^High^/CD56^Low^ and the graph in panel (**e**) represent the ratio of neutrophils to NK cells within the leukocyte population in ocular surface wash samples of eyes with different grades of KC. The graphs indicate the percentage of CD45^+^CD3^+^—Pan-T cells (**f**), CD45^+^CD3^+^CD56^+^ (Natural Killer T) cells (**g**) and CD45^+^CD3^+^γδTCR^+^ (gamma delta) T cells (**h**) within the leukocyte population in ocular surface wash samples of eyes with different grades of KC. Ctrls—Controls; FFKC—Forme fruste keratoconus; All-KC—all the grades of keratoconus combined; SEM—standard error of the mean; Controls (23 eyes), All-KC (51 eyes), FFKC (6 eyes), grade 1 KC (14 eyes), grade 2 KC (8 eyes), grade 3 or 4 (23 eyes); Bar graphs represent Mean ± SEM; *P < 0.05, **P < 0.01,***P < 0.001, ****P < 0.0001, Mann–Whitney test.

A significant increase in the proportions of total natural killer (NK) cells (CD45^+^CD66b^−^CD3^−^CD56^+^), CD56^Low^ NK cells or cytotoxic NK cells (CD45^+^CD66b^−^CD3^−^CD56^Low^) and CD56^High^ NK cells or increased cytokine-producing NK cells (CD45^+^CD66b^−^CD3^−^CD56^High^) was present in KC (across all grades) compared to controls (Fig. 2a–c). In addition, the proportion of CD56^High^ NK cells were significantly higher than CD56^Low^ NK cells in KC (grade-1 and grade-3/4) compared to controls (Fig. 2d). Based on the knowledge regarding the inter-regulatory property between neutrophils and NK cells^13^, we investigated the ratio of total neutrophils (CD45^+^CD66b^+^) and total NK cells (CD45^+^CD66b^−^CD3^−^CD56^+^) in the study cohort. Compared to controls, a significantly lower ratio of neutrophils/NK cells ratio was observed across the grades of KC (Fig. 2e). No significant difference in the proportions of T cells (CD45^+^CD3^+^) and NKT (CD45^+^CD3^+^CD56^+^) cells was observed between KC and controls (Fig. 2f,g). However, a significant increase in the proportion of gamma delta T cells (CD45^+^CD3^+^γδTCR^+^) was observed in KC compared to controls (Fig. 2h). A higher proportion of gamma delta T cells was observed, particularly in grade-2 (not significant) and grade-3/4 (P < 0.001) KC compared to controls (Fig. 2h).

### Ocular surface soluble factor profile in KC patients

**Figure 3:**
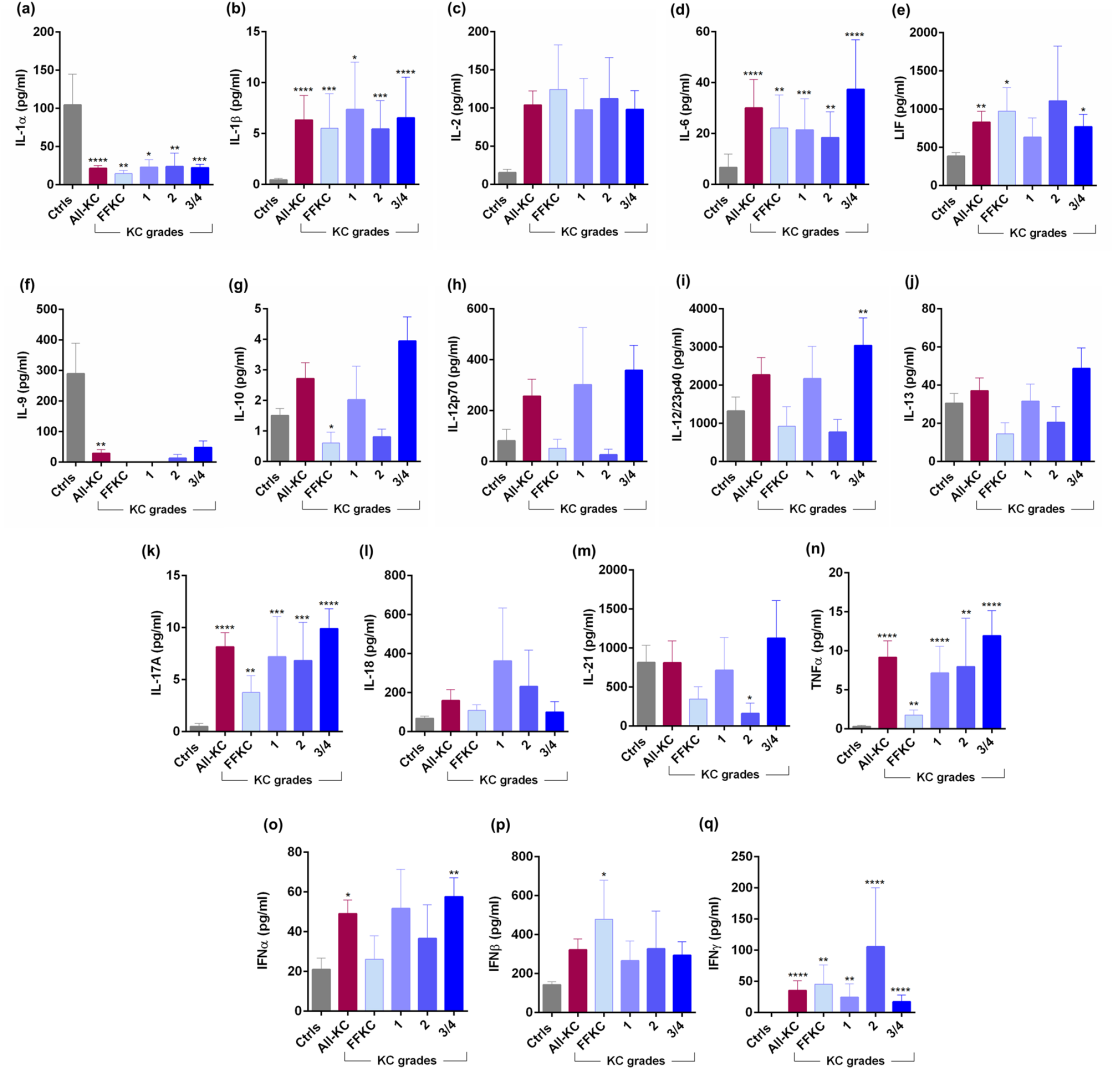
Tear fluid cytokines level in KC patients: The graphs indicate the concentration of IL-1α (**a**), IL-1β (**b**), IL-2 (**c**), IL-6 (**d**), LIF (**e**), IL-9 (**f**), IL-10 (**g**), IL-12p70 (**h**), IL-12/23p40 (**i**), IL-13 (**j**), IL-17A (**k**), IL-18 (**l**), IL-21 (**m**), TNFα (**n**), IFNα (**o**), IFNβ (**p**) and IFNγ (**q**) in the tear fluid of subjects with different grades of KC. Ctrls—Controls; All-KC—all the grades of keratoconus combined; FFKC—Forme fruste keratoconus; IL—interleukin; LIF—Leukemia inhibitory factor, an IL-6 class cytokine; TNFα—Tumour Necrosis Factor alpha; IFN—interferon; SEM—standard error of the mean; Controls (20 eyes), All-KC (41 eyes), FFKC (6 eyes), grade 1 KC (6 eyes), grade 2 KC (6 eyes), grade 3 or 4 (23 eyes); Bar graphs represent Mean ± SEM; *P < 0.05, **P < 0.01,***P < 0.001, ****P < 0.0001, Mann–Whitney test.

**Figure 4:**
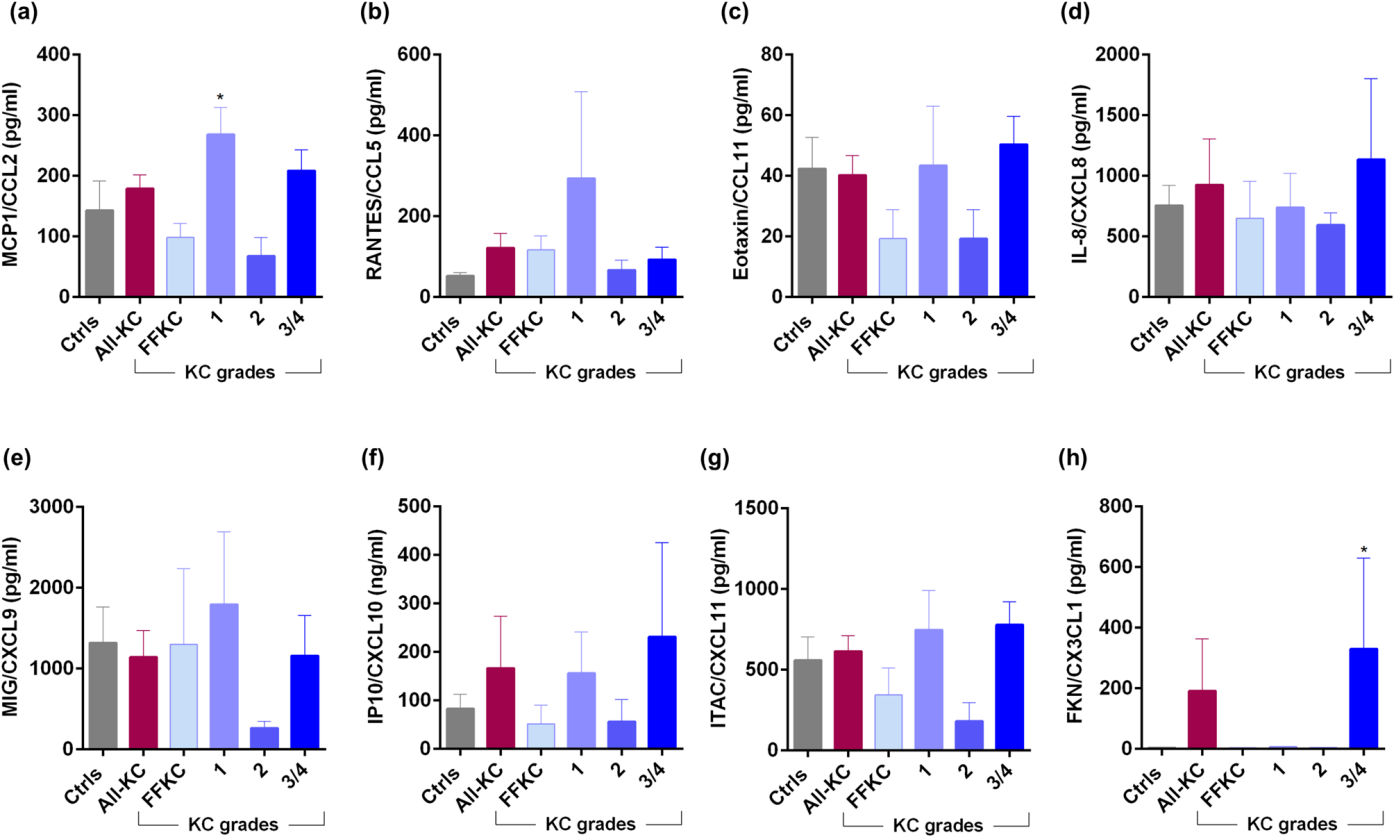
Tear fluid chemokines level in KC patients: The graphs indicate the concentration of MCP1/CCL2 (**a**), RANTES/CCL5 (**b**), Eotaxin/CCL11 (**c**), IL-8/CXCL8 (**d**), MIG/CXCL9 (**e**), IP10/CXCL10 (**f**), ITAC/CXCL11 (**g**) and FKN/CX3CL1 (**h**) in the tear fluid of subjects with different grades of KC. Ctrls—Controls; All-KC—all the grades of keratoconus combined; FFKC—Forme fruste keratoconus; FKN—fractalkine; IL—interleukin; MCP1—monocyte chemoattractant protein 1; MIG—monokine induced by gamma interferon; IP-10—Interferon gamma-induced protein 10; ITAC—Interferon-inducible T-cell alpha chemoattractant; RANTES—Regulated upon Activation, Normal T Cell Expressed and Presumably Secreted; SEM—standard error of the mean; Controls (20 eyes), All-KC (41 eyes), FFKC (6 eyes), grade 1 KC (6 eyes), grade 2 KC (6 eyes), grade 3 or 4 (23 eyes); Bar graphs represent Mean ± SEM; *P < 0.05, Mann–Whitney test.

**Figure 5:**
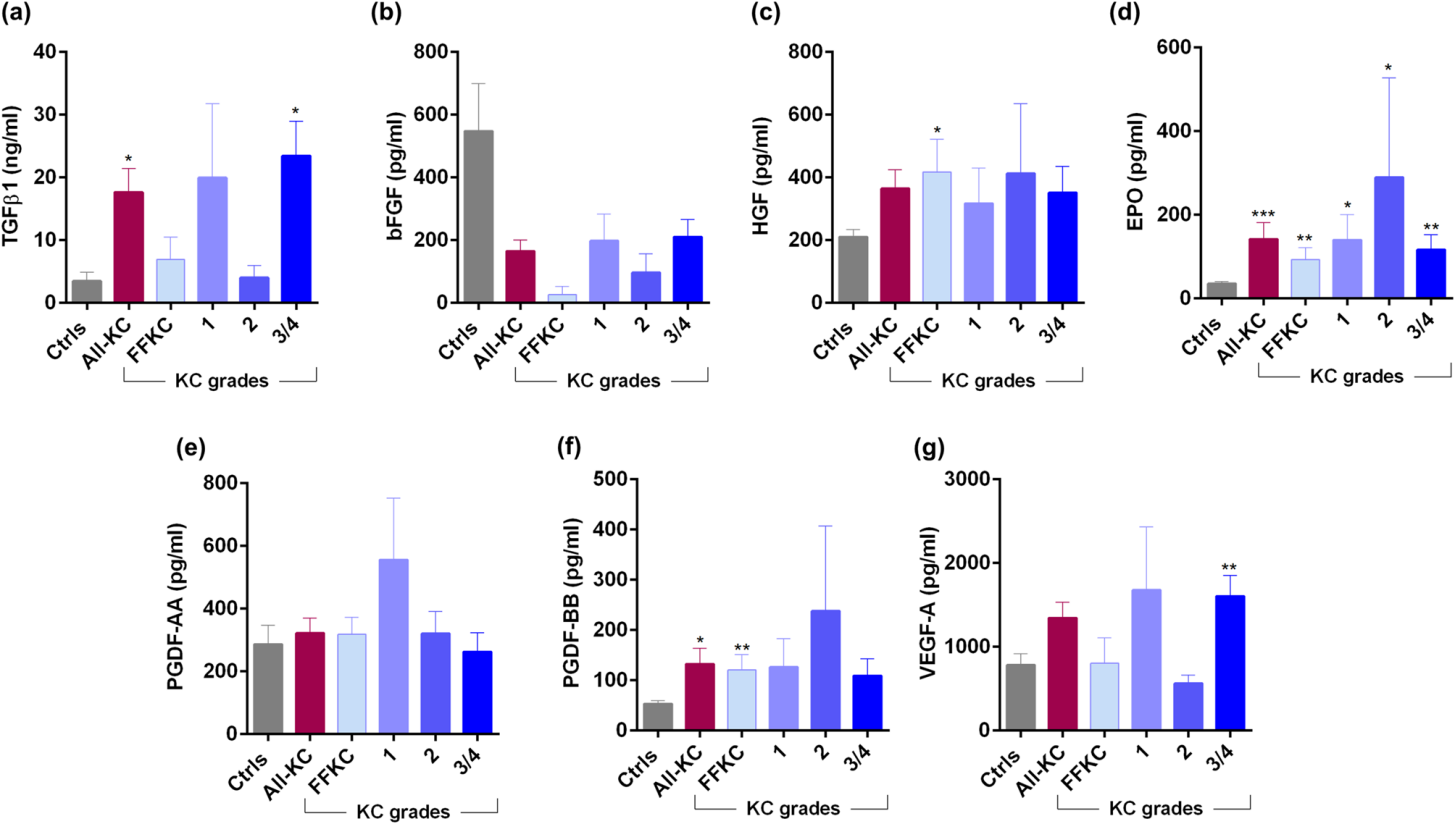
Tear fluid growth factors level in KC patients: The graphs indicate the concentration of TGFβ1 (**a**), bFGF (**b**), HGF (**c**), EPO (**d**), PDGF-AA (**e**), PDGF-BB (**f**) and VEGF-A (**g**) in the tear fluid of subjects with different grades of KC. Ctrls—Controls; All-KC—all the grades of keratoconus combined; FFKC—Forme fruste keratoconus; bFGF—basic fibroblast growth factor; HGF—Hepatocyte growth factor; EPO—erythropoietin; PDGF—Platelet-derived growth factor; TGFb1—Transforming Growth Factor Beta 1; VEGF—Vascular endothelial growth factor; SEM—standard error of the mean; Controls (20 eyes), All-KC (41 eyes), FFKC (6 eyes), grade 1 KC (6 eyes), grade 2 KC (6 eyes), grade 3 or 4 (23 eyes); Bar graphs represent Mean ± SEM; *P < 0.05, **P < 0.01,***P < 0.001, ****P < 0.0001, Mann–Whitney test.

**Figure 6:**
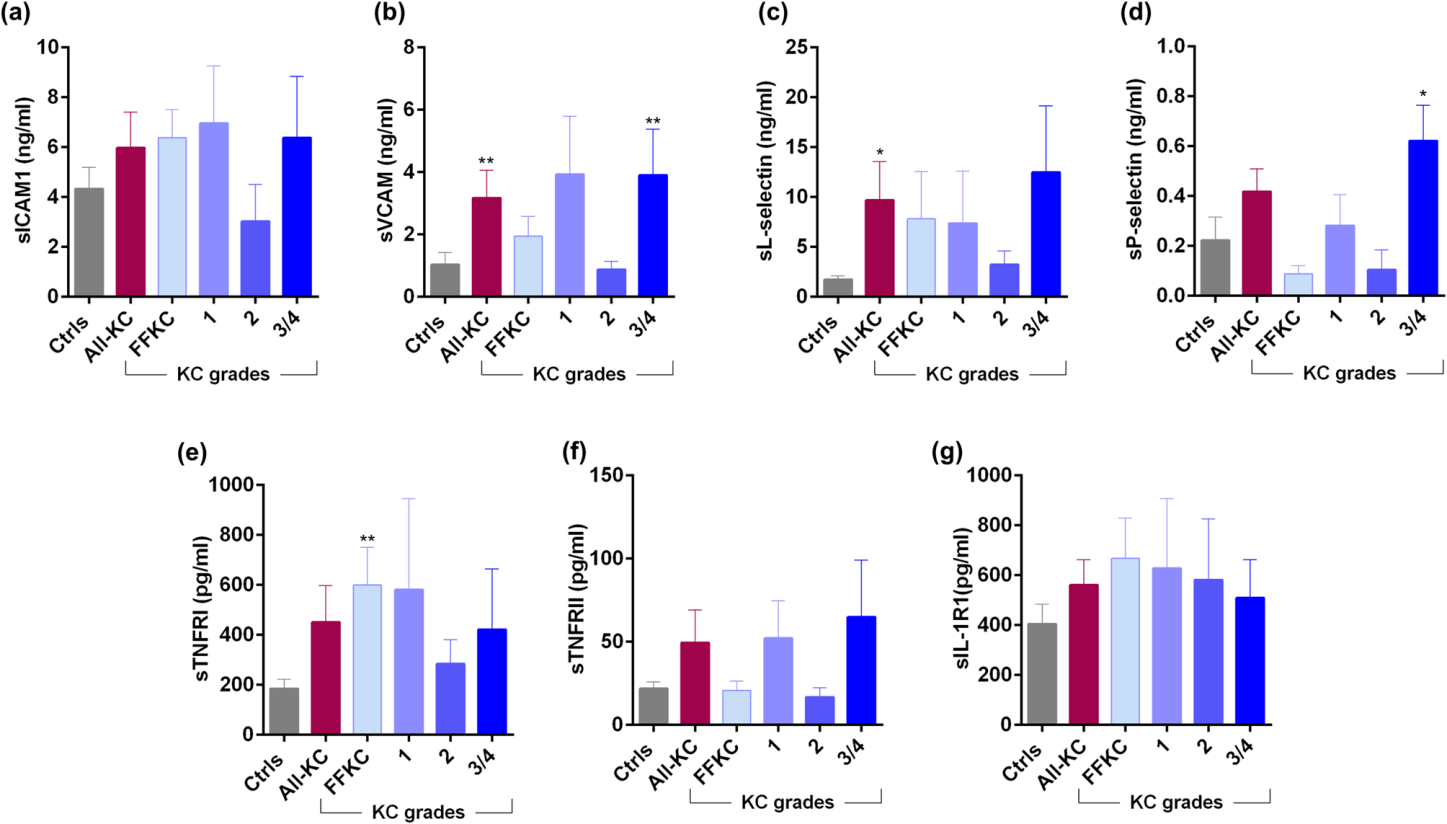
Tear fluid soluble cell adhesion molecules and soluble receptors level in KC patients: The graphs indicate the concentration of sICAM1 (**a**), sVCAM (**b**), sL-selectin (**c**), sP-selectin (**d**), sTNFRI (**e**), sTNFRII (**f**) and sIL-1R1 (**g**) in the tear fluid of subjects with different grades of KC. Ctrls—Controls; All-KC—all the grades of keratoconus combined; FFKC—Forme fruste keratoconus; sICAM1—Intercellular Adhesion Molecule 1; sVCAM—vascular cell adhesion molecule; sTNFRI—soluble tumor necrosis factor receptor 1; sTNFRII—soluble tumor necrosis factor receptor II; sIL-1R1—soluble interleukin 1 receptor type ISEM—standard error of the mean; Controls (20 eyes), All-KC (41 eyes), FFKC (6 eyes), grade 1 KC (6 eyes), grade 2 KC (6 eyes), grade 3 or 4 (23 eyes); Bar graphs represent Mean ± SEM; *P < 0.05, **P < 0.01,***P < 0.001, ****P < 0.0001, Mann–Whitney test.

**Figure 7:**
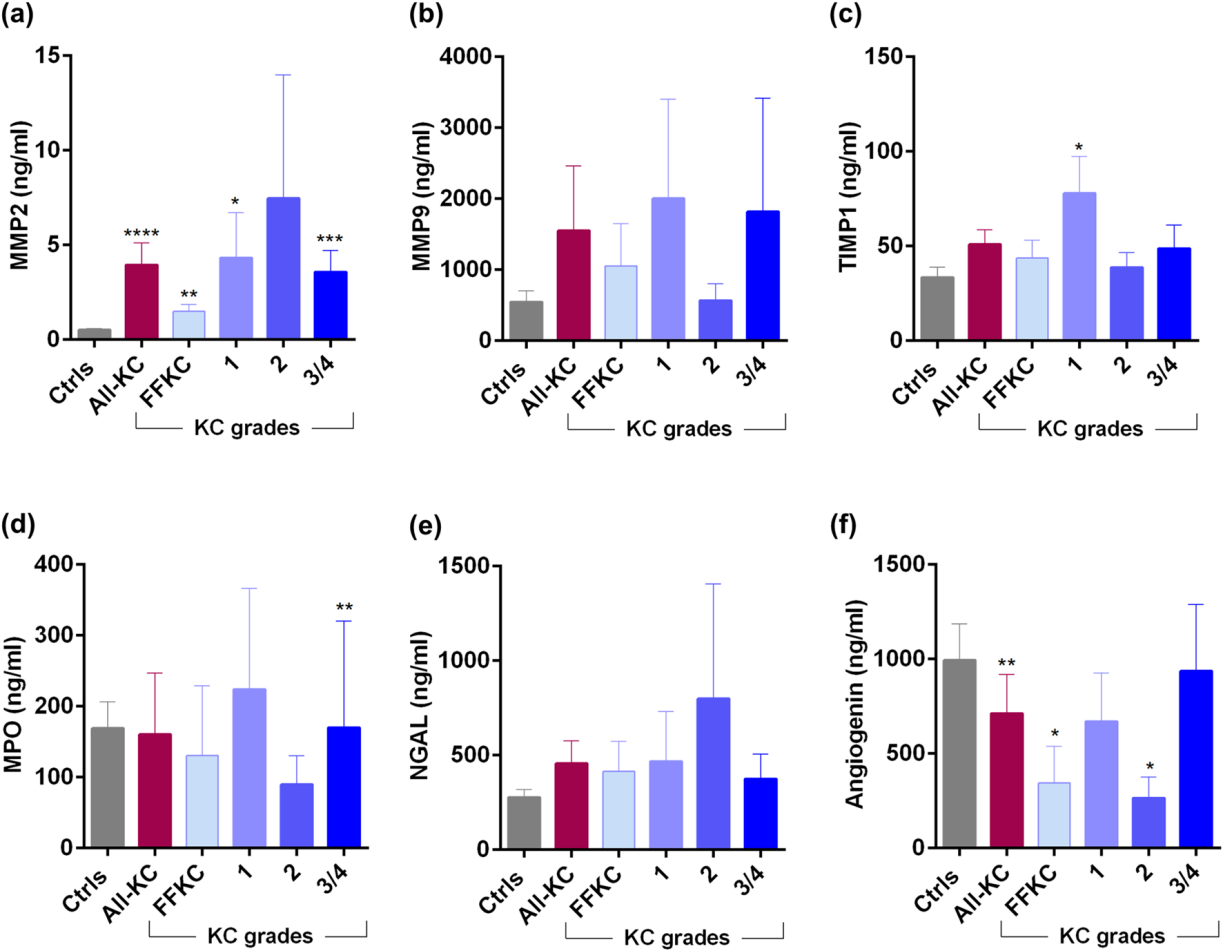
Tear fluid enzymes level in KC patients: The graphs indicate the concentration of MMP2 (**a**), MMP9 (**b**), TIMP1 (**c**), MPO (**d**), NGAL (**e**) and angiogenin (**f**) in the tear fluid of subjects with different grades of KC. Ctrls—Controls; All-KC—all the grades of keratoconus combined; FFKC—Forme fruste keratoconus; MMP—matrix metallopeptidases; MPO—myeloperoxidase; NGAL—neutrophil gelatinase-associated lipocalin; TIMP1—tissue inhibitor of metalloproteinases 1; SEM—standard error of the mean; Controls (20 eyes), All-KC (41 eyes), FFKC (6 eyes), grade 1 KC (6 eyes), grade 2 KC (6 eyes), grade 3 or 4 (23 eyes); Bar graphs represent Mean ± SEM; *P < 0.05, **P < 0.01,***P < 0.001, ****P < 0.0001, Mann–Whitney test.

**Figure 8:**
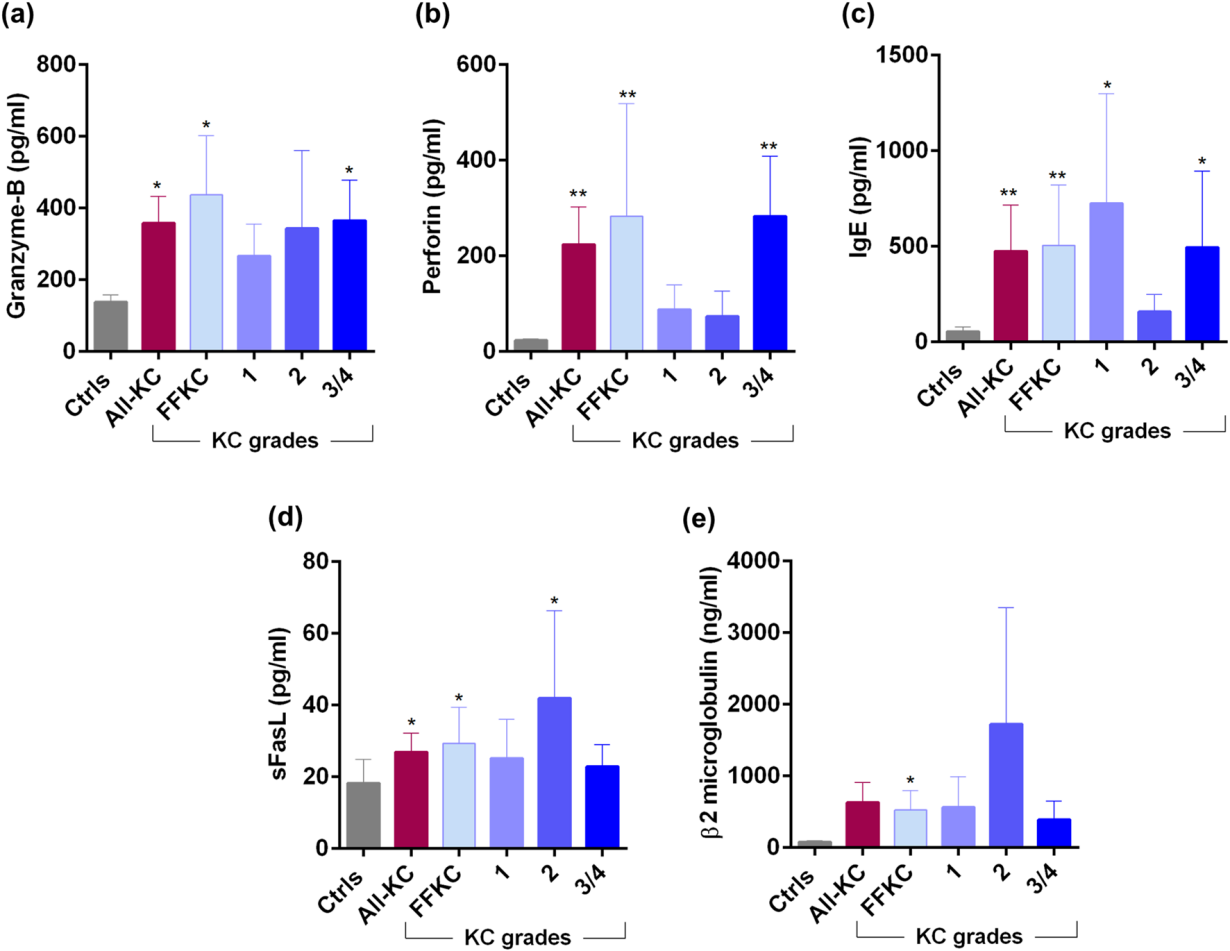
Tear fluid secreted factors level in KC patients: The graphs indicate the concentration of granzyme-B (**a**), perforin (**b**), IgE (**c**), sFasL (**d**) and β2 microgloublin (**e**) in the tear fluid of subjects with different grades of KC. Ctrls—Controls; All-KC—all the grades of keratoconus combined; FFKC—Forme fruste keratoconus; IgE—immunoglobulin E; sFasL—soluble Fas ligand; SEM—standard error of the mean; Controls (20 eyes), All-KC (41 eyes), FFKC (6 eyes), grade 1 KC (6 eyes), grade 2 KC (6 eyes), grade 3 or 4 (23 eyes); Bar graphs represent Mean ± SEM; *P < 0.05, **P < 0.01, Mann–Whitney test.

Marked changes in the levels of tear fluid cytokines (Fig. 3a–q), chemokines (Fig. 4), growth factors (Fig. 5), soluble cell adhesion molecules and soluble receptors (Fig. 6), enzymes (Fig. 7) and other secreted proteins (Fig. 8) was observed in KC compared to controls. *Cytokines:* The levels of interleukin (IL)-1β, IL-6, IL-17A, tumour necrosis factor-alpha (TNFα) and interferon-gamma (IFNγ) were significantly higher across all grades of KC compared to controls (Fig. 3b,d,k,n,q). Levels of IL-1α and IL-9 were significantly lower across all KC grades than controls (Fig. 3a,f). Significantly higher LIF (Leukemia inhibitory factor), an IL-6 class of cytokine, was observed in FFKC and grade-3/4 compared to controls (Fig. 3e). Compared to controls, the level of IL-10 was lower in early stages or lower grades of the disease and higher in grade-3/4 (Fig. 3g). The levels of IL-12p70 and IL-12/23p40 showed similar trends, but significantly higher levels of IL-12/23p40 was observed only in grade-3/4 KC samples (Fig. 3h,i). Though type 1 interferons (IFN), IFNα and IFNβ were higher in KC, they were not significantly higher across KC grades than controls, as was seen with IFNγ, a type 2 IFN (Fig. 3o–q). *Chemokines:* No significant differences in the levels of chemokines studied were observed between controls and KC (Fig. 4), except for monocyte chemoattractant protein 1 (MCP1/CCL2) and Fractalkine (CX3CL1) that exhibited significantly higher levels in grade-1 and grade-3/4, respectively (Fig. 4). *Growth factors:* Erythropoietin (EPO) was significantly higher across the grades of KC compared to control (Fig. 5). A significant increase in the levels of Transforming growth factor-beta 1 (TGFβ1) and vascular endothelial growth factor (VEGF) was observed in grade-3/4 KC compared to control (Fig. 5). Similarly, higher levels of hepatocyte growth factor (HGF) and platelet-derived growth factor-BB (PDGF-BB) were recorded in FFKC compared to control (Fig. 5). *Soluble cell adhesion molecules and soluble receptors:* Significantly higher levels of soluble vascular cell adhesion molecule (sVCAM) and soluble P-selectin were observed in grade-3/4 KC (Fig. 6), along with a significant increase in soluble tumour necrosis factor receptor 1 (sTNFRI) in FFKC compared to controls (Fig. 6). *Enzymes:* Significantly higher levels of matrix metalloproteinase 2 (MMP2) was observed across the grades of KC, and increased levels of MMP9 (although not statistically significant) was observed compared to controls (Fig. 7). Interestingly, angiogenin, a RNase, was significantly lower in FFKC and grade-2 KC compared to controls (Fig. 7). *Other secreted factors:* Granzyme-B, perforin and soluble Fas ligand (sFasL) were significantly higher in FFKC and grade-3/4 than controls (Fig. 8). Immunoglobulin E (IgE) was significantly higher in all grades except grade-2 KC, and β2 microgloublin was significantly higher in FFKC than controls (Fig. 8).

**Figure 9:**
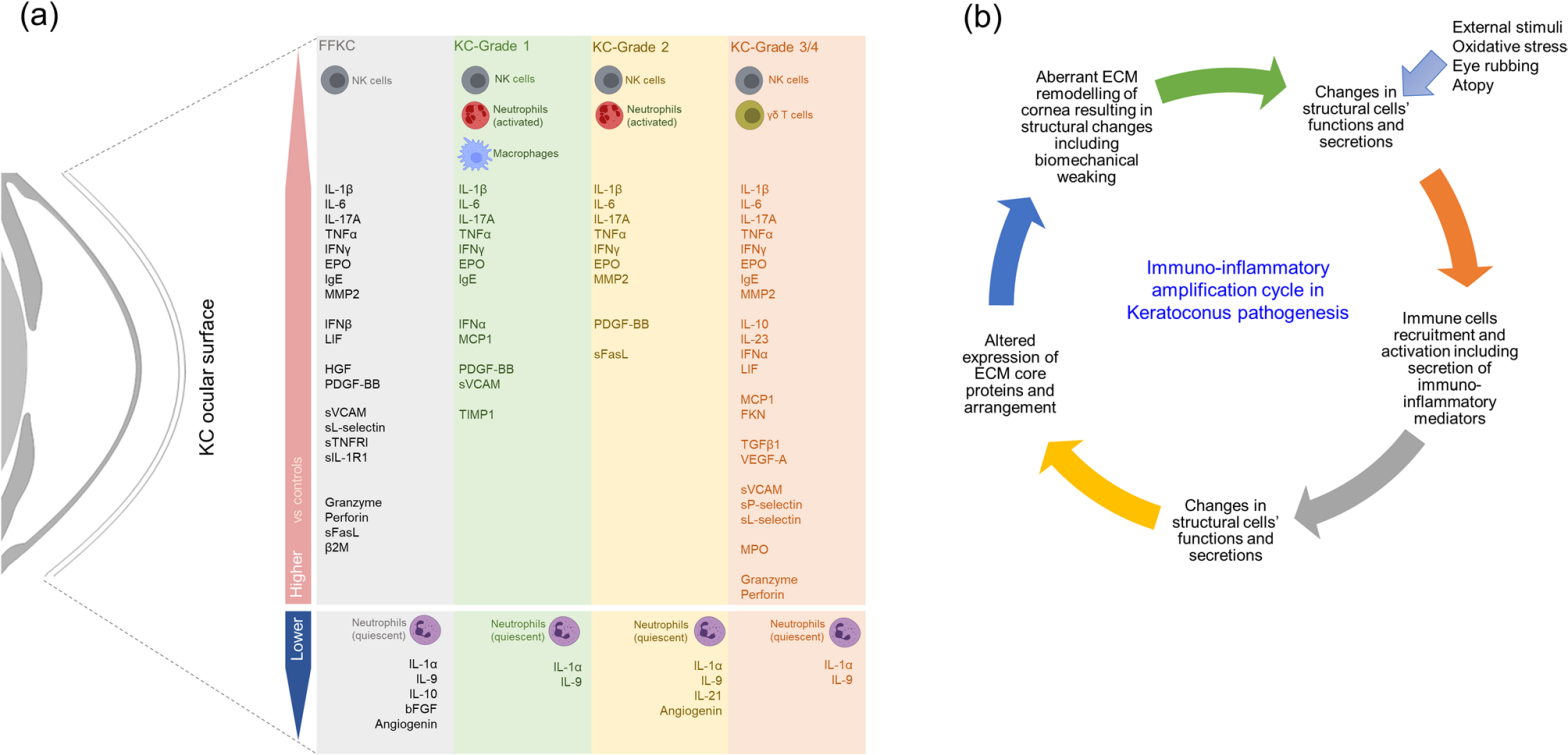
Ocular surface immuno-inflammatory status in KC: (**a**) The schematic represents the immune cell subtypes on the ocular surface of KC patients (across different grades of the disease) that were significantly different increased or decreased compared to controls. The schematic also indicates the various tear fluid soluble factors whose levels were significantly altered in KC (across different grades of the disease) compared to controls. (**b**) Represents the hypothetical immuno-inflammatory amplification cycle in keratoconus pathogenesis. External stimuli including physical and/or biological stimuli, oxidative stress, eye rubbing, etc., can stimulate the corneal structural cells such as the epithelium and keratocytes to secrete various active biologically active factors, including those with inflammatory and chemoattract properties. This will facilitate the recruitment and activation of immune cells on the ocular surface. The activation of immune cells might result in additional secretion of immune- and inflammatory mediators which can adversely impact the homeostasis and function of the corneal structural cells. This can result in aberrant extra-cellular matrix (ECM) remodeling due to decreased collagen synthesis, reduction in endogenous cross-linking enzyme and increased degradation of ECM proteins via proteases. Thus, causing structural and biomechanical changes characteristics of KC. The function of corneal epithelium and keratocytes will further be influenced by changes in the biophysical cues due to altered ECM remodeling, thus contributing to disease progression in KC.

Correlation between the proportion of a specific immune cell subset and its respective secretory marker in the tear fluid was determined to validate the findings. A positive association of NK cell proportion with granzymes and perforin levels; neutrophil proportion with myeloperoxidase (MPO) and neutrophil gelatinase-associated lipocalin (NGAL) levels; and gamma delta T cell proportion with TNFα, IFNγ and IL-17A levels were observed in the study cohort (Table 1). The overall immune cell subsets proportion and soluble factor profile changes across the different grades of KC is summarized in Fig. 9.

**Table 1:** Association between ocular surface immune cell type proportion and their characteristic secretory factors levels in the tear fluid of study subjects.

Soluble factors	r	P value
**(a) CD56**^**Total**^** cells**		
Granzyme-B	0.334	0.009
Perforin	0.382	0.002
**(b) CD66b**^**Total**^** cells**		
MPO	0.269	0.036
NGAL	0.325	0.011
**(c) CD3**^**+**^**γδTCR**^**+**^**T cells**		
TNFα	0.411	0.001
IFNγ	0.383	0.002
IL-17A	0.423	0.001

r = Spearman rank correlation coefficient.

### Relationship among ocular surface immune cell subsets proportions, tear fluid soluble factor levels, keratometry/pachymetry indices, allergy and eye rubbing in KC patients

A positive correlation was observed between gamma delta T cells proportions and keratometry indices such as K1, K2 and Kmean (Table 2). Further, a negative correlation was observed between gamma delta T cells proportions and pachymetry indices such as central corneal thickness and thinnest corneal thickness (Table 2). A positive correlation between pan-T cell proportion and Kmean, and a negative correlation between pan-T cell proportion and thinnest corneal thickness was also observed (Table 2). Interestingly, a positive association between activated neutrophil proportion and central corneal thickness and thinnest corneal thickness was noted (Table 2). The levels of IL-13, IL-17A, TNFα, Eotaxin and sP-selectin exhibited a positive association with one or more of the four keratometry indices such as K1, K2, Kmean and Kmax (Table 3). lL-12p70 and TGFβ1 levels were also positively related (near statistical significance) with K2 and Kmax (Table 3). On the contrary, the levels of sTNFRI, NGAL and EPO was observed to have a negative relationship with one or more of the keratometry indices (Table 3). Interestingly, a positive association was observed between the levels of IL-10 and keratometry indices, despite a negative association with pachymetry indices (Table 3).

**Table 2:** Association of ocular surface immune cell subset proportions with corneal keratometry and pachymetry indices of KC patients.

Proportion of ocular surface immune cell subsets	K1 (D)		K2 (D)		Kmean (D)		Kmax (D)		CCT (μm)		TCT (μm)	
	r	P value	r	P value	r	P value	r	P value	r	P value	r	P value
**Total leukocytes**												
CD45^+^ cells	− 0.018	0.901	0.082	0.568	0.035	0.809	0.110	0.441	0.088	0.541	0.075	0.600
**Neutrophil subsets**												
CD66b^Total^ cells	− 0.037	0.794	− 0.044	0.758	− 0.047	0.745	− 0.015	0.919	0.156	0.273	0.124	0.386
CD66b^Low^ cells	0.203	0.152	0.137	0.339	0.171	0.229	0.095	0.508	− 0.111	0.438	− 0.109	0.447
CD66b^High^ cells	− 0.257	0.069	− 0.240	0.090	− 0.252	0.074	− 0.210	0.139	0.336	0.016	0.312	0.026
CD66b^High^/CD66b^Low^ cells ratio	− 0.236	0.096	− 0.218	0.125	− 0.227	0.110	− 0.163	0.254	0.254	0.073	0.274	0.052
**Macrophages**												
CD163^+^ cells	− 0.094	0.512	− 0.120	0.401	− 0.101	0.481	− 0.134	0.349	0.082	0.565	0.081	0.574
**Natural killer (NK) cell subsets**												
CD56^Total^ cells	0.014	0.923	− 0.001	0.995	0.012	0.933	− 0.005	0.974	− 0.238	0.092	− 0.236	0.095
CD56^Low^ cells	− 0.063	0.662	− 0.067	0.643	− 0.063	0.662	− 0.066	0.643	− 0.190	0.182	− 0.190	0.181
CD56^High^ cells	0.130	0.364	0.046	0.749	0.098	0.496	0.052	0.715	− 0.089	0.535	− 0.072	0.617
CD56^High^/CD56^Low^ NK cells ratio	0.171	0.235	0.168	0.244	0.172	0.233	0.144	0.320	− 0.139	0.337	− 0.149	0.302
CD66b^+^/CD56^+^ cells ratio	− 0.036	0.802	− 0.034	0.813	− 0.043	0.762	− 0.007	0.960	0.199	0.162	0.169	0.235
**T cell subsets**												
CD3^+^T cells	0.271	0.055	0.273	0.053	0.277	0.049	0.221	0.119	− 0.272	0.054	− 0.313	0.026
CD3^+^CD56^+^ T (NKT) cells	0.016	0.913	0.057	0.700	0.080	0.588	0.044	0.764	− 0.101	0.493	− 0.093	0.532
CD3^+^γδTCR^+^ T cells	0.345	0.013	0.324	0.020	0.344	0.014	0.254	0.072	− 0.307	0.029	− 0.300	0.032

r = Spearman rank correlation coefficient.

**Table 3:** Association of tear fluid soluble factor levels with corneal keratometry and pachymetry indices of KC patients.

Soluble factors	K1 (D)		K2 (D)		Kmean (D)		Kmax (D)		CCT (um)		TCT (um)	
	r	P value	r	P value	r	P value	r	P value	r	P value	r	P value
IL-10	0.492	0.001	0.513	0.001	0.504	0.001	0.518	0.001	− 0.300	0.057	− 0.321	0.041
IL-12p70	0.190	0.235	0.264	0.096	0.223	0.161	0.291	0.065	− 0.014	0.929	− 0.031	0.846
IL-13	0.334	0.033	0.334	0.033	0.331	0.034	0.325	0.038	− 0.120	0.456	− 0.128	0.427
IL-17A	0.307	0.051	0.340	0.029	0.330	0.035	0.345	0.027	− 0.110	0.493	− 0.140	0.383
TNFα	0.388	0.012	0.402	0.009	0.404	0.009	0.410	0.008	− 0.211	0.186	− 0.245	0.122
sTNFRI	− 0.306	0.052	− 0.325	0.038	− 0.317	0.043	− 0.306	0.051	0.184	0.249	0.211	0.185
Eotaxin	0.229	0.150	0.303	0.054	0.260	0.100	0.331	0.034	− 0.083	0.606	− 0.093	0.564
NGAL	− 0.242	0.127	− 0.316	0.044	− 0.273	0.084	− 0.313	0.046	0.181	0.258	0.200	0.210
EPO	− 0.207	0.194	− 0.270	0.088	− 0.236	0.137	− 0.321	0.041	0.163	0.309	0.183	0.253
sP-selectin	0.424	0.006	0.385	0.013	0.391	0.012	0.382	0.014	− 0.240	0.130	− 0.234	0.141
TGFβ1	0.201	0.208	0.279	0.077	0.236	0.138	0.301	0.056	− 0.032	0.841	− 0.054	0.739

r = Spearman rank correlation coefficient.

No significant difference in the ocular surface immune cell subset proportions and tear fluid soluble factor levels was observed between FFKC and contralateral KC eyes of different grades (Supplementary Tables 1 and Table 2). Since, allergy is strongly associated with KC, the influence of mild ocular allergy, history of systemic allergy and eye rubbing on the immune cell profile and soluble factor profile were also investigated. KC subjects with ongoing severe ocular or systemic allergy were excluded from the study. Hence, the subjects with mild ocular allergy included in the study are those who presented with KC along with mild or inactive conjunctival papillae and without any signs of congestion or redness. Immune cell subset proportions, particularly those that were significantly altered in KC compared to controls, were not observed to be significantly different between KC patients with and without history of systemic allergic disease (Supplementary Table 3), with and without mild ocular allergy (Supplementary Table 4) and, with and without history of eye rubbing (Supplementary Table 5). Significantly higher tear fluid levels of IL-1α, IL-9, IL-10, IL-13, TNFα, sVCAM, sTNFRII and IgE were observed in KC subjects with history of systemic allergy compared to those without (Supplementary Table 6). Significantly higher levels of cytokines (IL-2, IL-12/23p40, IL-12p70, IL-13, IL-17A, IFNα), chemokines (MIG/CXCL9; ITAC/CXCL11), growth factors (TGFβ1, EPO, VEGF) and enzyme (MPO and angiogenin); and a significant reduction in NGAL was also observed in KC subjects with a history of eye rubbing (Supplementary Table 7). A similarly profile was observed in KC patients with history of eye rubbing, since almost all those who presented with mild allergy reported history of eye rubbing. Significantly higher levels of cytokines (IL-2, IL-6, IL-12/23p40, IL-12p70, IL-13, IFNα), chemokines (Eotaxin/CCL11; IL-8/CXCL8; MIG/CXCL9; ITAC/CXCL11), growth factors (TGFβ1, EPO, VEGF) and enzyme (angiogenin); and a significant reduction in enzymes such as TIMP1 and NGAL was also observed in KC subjects with a history of eye rubbing (Supplementary Table 8).

## Discussion

Keratoconus features global ultrastructural and molecular changes that result in focal corneal steepening and epithelial thinning and subsequent decrease in visual acuity and quality of life^3^. Extra-cellular matrix remodelling is a dynamic process that involves synchronized deposition, arrangement and degradation of ECM proteins rendering structural integrity to organs and tissues, thus influencing their functional status. The formation and assembly of collagens, one of the main ECM core proteins, is compromised in the KC cornea^14–19^. Further, the most dominant collagen crosslink type, lysinonorleucine, is diminished in KC^20^, along with a reduction in the expression^16,19,21,22^ and activity^16^ of a key endogenous collagen crosslinking enzyme, Lysyl oxidase (LOX). An imbalance in the levels of proteolytic enzymes and their negative regulators that can adversely impact ECM remodelling was observed in KC. The expression and activity of proteolytic enzymes are also increased in KC. These include matrix metalloproteinases (MMPs), cathepsins, collagenase, gelatinase, peptidase and heparinase^16,19,23–36^. In parallel, the expression of endogenous regulators of proteolytic enzymes is also reduced in KC, such as alpha 1-proteinase inhibitor, alpha 2-macroglobulin, and tissue inhibitor of matrix metalloproteinase 1. Prolidase activity that facilitates collagen turnover or synthesis is also reduced in KC^35,37–39^. Various factors including immune and inflammatory mediators can impact the above-mentioned key ECM remodelling components.

The current study demonstrates an altered state of immune cells and inflammatory mediators on the ocular surface of KC patients in a grade specific manner (Fig. 9a). IL-1β, IL-6, IL-17A, TNFα, IFNγ, IgE, MMP2, EPO, PDGF-BB, sVCAM, sL-selectin, granzyme-B and perforin were observed to be significantly increased compared to controls in two or more of KC grades. This finding is consistent with earlier observations reporting increased levels of IL-1β^26,40–42^, IL-6^24,26,30^, TNFα^41^, IL-17A^40,43^, IFNγ^44^ and MMP2^26^ in the tear fluid of KC patients. The observed variation in tear fluid expression of MMP9^24–33,45^, IL-8^26,33,46^, IL-13^40^, IL-21^40^, IL-23^40^, IFNα^40^, MCP1^40^ and CCL5^31,32,44^ between the current study and previous reports, may be attributed to differences in sample size and stage of KC investigated in the cohort.

Allergy, atopy and eye rubbing are suggested to play an essential role in the pathogenesis of KC and one of the risk factors for worsening of the disease^47,48^. Eye rubbing is associated with IgE driven conditions such as allergy or atopy with some KC patients presenting with elevated serum IgE^47^. In the current study we report significantly higher levels of tear fluid IgE in KC patients, particularly in those with history of systemic allergy further strengthening the relationship between IgE and KC. In addition, the levels of TNFα and sVCAM observed to be higher in KC were also specifically higher in those KC patients with history of systemic allergy. Tear fluid levels of IL-2, IL-12/23p40, IL-12p70, IL-13, IFNα, MIG/CXCL9; ITAC/CXCL11, TGFβ1, EPO, VEGF, angiogenin and NGAL were similarly dysregulated in those KC patients with mild ocular allergy and history of eye rubbing. In addition, previous studies report increase tear fluid factors such as IL-6 and TNFα levels following eye rubbing despite the absence of allergy^49^. This suggests, there are specific inflammatory factors that are dysregulated in KC that may not be associated with allergy response. Therefore, irrespective of the association of inflammatory factors with other related co-morbidities, it is critical to identify them, especially those that are dysregulated and can alter the ECM remodelling. In addition, the role of allergy-associated factors needs to be dissected further to determine their contribution (either redundant or additive) in corneal ECM remodelling in conjunction with non-allergy associated inflammatory factors.

IL-1β is known to increase the expression of MMPs^50,51^, induce ECM degradation^52^, and decrease the expression of lysyl oxidase^50,53^ and collagens^53^ in other cells and tissues. Similarly, IL-6^54^, IL-17A^55^, TNFα^56^ and IFNγ^57,58^ induce the expression of MMPs and inhibit the production of collagen in various cells and tissues. The levels of TGFβ1, a potent pro-fibrotic factor, were higher in tear fluid of KC patients^40^, however, the current study observed a significant increase in TGFβ1 only in the highest grade of KC. This finding is in line with fibrotic changes observed in advanced KC. Similarly, anti-inflammatory factor, IL-10 was reported to be either increased ^26,40,41^ similar to the higher grade of KC in the current study or decreased^32,43,59^ in tear fluid of KC patients, similar to early grade observation in the present study. IL-9, which is significantly reduced in KC, is a negative regulator of IL-17A in immune cells^60^, possibly indicating one of the mechanisms underlying increased IL-17A in KC patients.

Erythropoietin (EPO), a key growth factor regulating erythropoiesis, modulates ECM remodelling by stimulating collagen synthesis, TIMPs and inhibiting MMPs in other tissues^61–63^. The role of EPO is supported by the negative association between EPO and Kmax in the current study (Table 3). Therefore, the elevated EPO levels in KC could play a compensatory or protective role in KC. Granzyme-B and perforin produced predominantly by NK cells could impact ECM remodelling in the cornea. Granzyme-B is known to degrade several ECM components. In a perforin-dependent or independent manner, it facilitates the death of structural cells by detachment-mediated cell death—anoikis^64^. The elevated levels of granzyme-B and perforin and increased cytokine-producing NK cells in KC suggest an alleged detrimental role in KC. Beta-2 microglobulin, shown to increase in several inflammatory conditions^65,66^ also increased in our KC cohort, can indirectly impact ECM remodelling. PDGF-BB elevated in early stages of KC is shown to induce the expression of ECM proteins such as collagens in vitro^67^.

Neutrophils are the first line of innate immune defence. Neutrophils migrate to the site of injury or infection to render protection, repair and restore homeostasis^13,68^. Aberrant neutrophil activation is often associated with pathology in a variety of tissues^13,68^. Although neutrophil–lymphocyte ratio (NLR) in the circulation of KC patients was reported to be increased^69,70^, the proportion varied between studies and one study reported no significant increase in the NLR in KC patients^71^. Hence, studying the local neutrophil status, such as that on the KC patient's ocular surface, would prove relevant and valuable. Increased proportions of activated neutrophils have been observed on the ocular surface of KC patients. However, a favourable association was observed between these cells and corneal pachymetry indices (Table 2). Moreover, a similar relationship was observed between the neutrophil secreted product NGAL and corneal keratometry indices (Table 3).

Natural killer (NK) cells are also part of the innate immune system. These lymphocytes are involved in the first line of defence against infected and malignant cells by directly killing the cells or activating other immune cells by secreting cytokines and chemokines. NK cells are present on mucosal surfaces, including the cornea and conjunctiva^72–74^. In addition, to a significantly higher proportion of NK cells present on the ocular surface of KC patients, a close to significant negative relationship was observed between NK cell proportion and corneal pachymetry indices (Table 2). The opposite association between neutrophils and NK cells with corneal pachymetry suggests a possible counter-regulatory role of these cells on the ocular surface of KC patients. Decreased neutrophils-NK cell ratio in KC compared to controls is suggestive of neutrophil–NK cell proportion imbalance with increased proportion of NK cells over the neutrophils on the ocular surface of KC patients. Interestingly, in DED, the neutrophil–NK ratio is significantly increased^10^, suggesting the dominant presence of neutrophils on the ocular surface of DED patients. The interplay between neutrophils and NK cells^13^ on the ocular surface and their inter-regulatory dynamics are altered in different disease processes.

Gamma delta T cells, innate-like lymphoid cells are commonly present in mucosal surfaces contributing to both homeostasis and disease^75–77^, and their numbers are increased in inflamed ocular surface mucosa^78,79^. Gamma delta T cells produce various cytokines, including IL-17A, IFNγ and TNFα^77,80^. In the current study, a significant increase in gamma delta T cells, along with a significant increase in -17A, IFNγ and TNFα, was observed in KC. The association between the proportion of gamma delta T cells, inflammatory cytokines (Table 1c), and corneal keratometry/pachymetry indices (Table 2) support their role in KC pathogenesis.

The current findings confirm that the various grades of KC are characterized by distinct changes in immune cell subset proportions on the ocular surface and tear fluid inflammatory factors that correlate to corneal keratometry and pachymetry indices. Grade-specific differences in immuno-inflammatory factors observed in the current study could be related to disease progression, the compensatory reparative response and the associated expression of core ECM proteins in the KC cornea^81^. Limitations of the study are the small sample size of patients in the earlier grades of KC and the collection of superficial and trafficking immune cells rather than cells embedded deep in the mucosal tissue.

The current study describes ocular surface immuno-inflammatory landscape in KC and provides important information pertaining to the biologically active components on the ocular surface of KC. The following are the summary of the key novel findings (1) Proportion of ocular surface immune cell profile status (11 subsets) in KC patient eyes will have very profound impact on our understanding of KC. (2) Given the fact that understanding of KC evolved from “non-inflammatory disease” to the current general agreement that it does indeed have a strong inflammatory component, current data on immune cells and their correlation to tear inflammatory factors provides the first concrete evidence of an immunologically relevant source of inflammation in KC patients. (3) NK cells were found to be elevated significantly across the groups, while neutrophils were not. Note that NK cell dysregulation and its correlation with secreted factors granzymes and perforins (which are known to be secreted by NK cells, but not reported in KC literature) shown in the study have not been reported previously in the ocular surface field. This suggests a causal relationship between the secretory profile of the dysregulated NK cells in KC eyes. (4) The ratio of neutrophils to NK cells that is opposite in KC compared to DED is an entirely new concept in the field which can be harnessed in designing treatment options for KC. (5) T-cells and γδTCR+T cells in particular were significantly positively correlated with clinical indices K-mean while negatively correlated with corneal thickness. Further this cell type correlated significantly with specific inflammatory cytokines in patients. (6) 50 different soluble factors across different functional class (cytokines, chemokines, soluble cell adhesion molecules, soluble receptors, growth factors, enzymes) including IgE were measured in tear fluid across different of grades of KC along with the matched immune cells is a comprehensive and important addition to KC knowledge base. In addition to well-known inflammatory factors, we also identified novel factors significantly altered on the ocular surface in KC. Irrespective of the source of the immune-inflammatory components it is very pertinent to understand and study the newly observed deregulated factors (Fig. 9) on the ocular surface in KC pathogenesis, disease monitoring and treatment planning. Our findings support the value of non-invasive methods to interrogate the immune status of the ocular surface to monitor and prevent disease progression and guide targeted therapies. In future, in vitro mechanistic validation and a prospective clinical study focused on the relationship between ocular surface immuno-inflammatory factors and corneal changes documented by keratometry, pachymetry and ultrastructure (e.g., polarization-sensitive optical coherence tomography) are needed to determine the functional relationships between these factors and KC pathogenesis.

## Supplementary Information

Supplementary Legends.

Supplementary Figure 1.

Supplementary Figure 2.

Supplementary Table 1.

Supplementary Table 2.

Supplementary Table 3.

Supplementary Table 4.

Supplementary Table 5.

Supplementary Table 6.

Supplementary Table 7.

Supplementary Table 8.
