# Supplementary material for: Keratoconus patients exhibit a distinct ocular surface immune cell and inflammatory profile
Source: Sci Rep. 2021 Oct 22;11:20891. doi: 10.1038/s41598-021-99805-9 (PMC8536707; doi:10.1038/s41598-021-99805-9)
Supplement: Supplementary file 1 — Supplementary Legends. [file 41598_2021_99805_MOESM1_ESM.docx]

**SUPPLEMENTARY FIGURE LEGENDS**

**Supplementary Figure 1. Status of corneal keratometry and pachymetry indices in keratoconus patients:** The graphs indicate (a) flat keratometry – K1, (b) steep keratometry – K2, (c) mean keratometry – Kmean, (d) maximum keratometry – Kmax, (e) central corneal thickness and (f) thinnest corneal thickness in KC patients. CCT – central corneal thickness; FFKC – Forme fruste keratoconus; D – diopter; KC – keratoconus; TCT – thinnest corneal thickness; SEM - standard error of the mean; FFKC (6 eyes), grade 1 KC (14 eyes), grade 2 KC (8 eyes), grade 3 or 4 (23 eyes); Bar graphs represent Mean±SEM; **P<0.01, ***P<0.001, ****P<0.0001, Mann-Whitney test.

Supplementary Figure 2. Gating strategy to phenotype ocular surface immune cell subsets in study subjects with and without keratoconus: Representative images shows gating strategies used to determine the immune cell populations (specifically leukocytes, neutrophils, macrophages, natural killer cells, T cells, gamma delta T cells and NKT cells) in the ocular surface wash samples from subjects with and without KC. The cells from respective samples were stained for different cell surface markers-specific fluorochrome-conjugated antibodies and serial gating strategies (marked with arrows) were performed. (**i**) The marked region CD45 (APC-H7) versus Side Scatter (SSC) represents the leukocytes population. (**ii**) The top right quadrant in this panel indicates cells positively stained for both CD66b (AF647) and CD45 (APC-H7). They represent neutrophils present among the CD45^+^ cells. The quadrant is further subdivided into two types based on the expression or signal strength of CD66b (AF647) into CD66b^High^ and CD66b^Low^ representing the activated and quiescent neutrophil populations, respectively. (**iii**) The upper right quadrant represents macrophages, the cells that are positively stained for both and CD163 (FITC) and CD45 (APC-H7). (iv) The top left quadrant in this panel indicates CD45 (APC-H7) positive cells that are stained for CD56 (PE-Cy7) but not CD66b (AF647). These cells represent Natural Killer (NK) cells present among the CD45^+^ cells. The quadrant is further subdivided into two based on the expression or signal strength of CD56 (PE-Cy7) into CD56^High^ and CD56^Low^ representing the enhanced cytokine producing less cytotoxic and cytotoxic forms of NK populations, respectively. (**v**) The upper right quadrant represents cells that are positively stained for both and CD3 (PE) and CD45 (APC-H7). These cells are categorized as pan-T cells among the CD45^+^ cells. (vi) The upper right quadrant in this panel indicates CD45 (APC-H7) positive cells stained for both γδTCR (PerCP-Cy5.5) and CD3 (PE) and they represent gamma delta T (γδT) cells. (**vii**) The upper right quadrant represents CD45 (APC-H7) positive cells stained for both and CD56 (PE-Cy7) and CD3 (PE) and represent Natural Killer T (NKT) cells. The numbers of specific immune cells along with the number of leukocytes were used to compute the percentage of each immune cell subsets within the ocular surface leukocyte population for each study subject.
