# Supplementary material for: Keratoconus patients exhibit a distinct ocular surface immune cell and inflammatory profile
Source: Sci Rep. 2021 Oct 22;11:20891. doi: 10.1038/s41598-021-99805-9 (PMC8536707; doi:10.1038/s41598-021-99805-9)
Supplement: Supplementary file 4 — Supplementary Table 1. [file 41598_2021_99805_MOESM4_ESM.docx]

**Supplementary Table 1:** **Ocular surface immune subset proportions in study subjects with** **forme fruste keratoconus in one eye and KC in the contralateral eye**

| **Ocular surface immune cell subset proportions** | **FFKC (n=6)** | | | **KC (n=6)** | | | **P value** |
| --- | --- | --- | --- | --- | --- | --- | --- |
|  | Mean | Stdev | SEM | Mean | Stdev | SEM |  |
| CD45^+^ cells | 35.8 | 35.0 | 14.3 | 44.0 | 29.4 | 12.0 | 0.589 |
| CD66b^Total^ cells | 14.9 | 11.7 | 4.8 | 18.7 | 13.7 | 5.6 | 0.589 |
| CD66b^Low^ cells | 12.8 | 9.3 | 3.8 | 13.9 | 8.2 | 3.3 | 0.818 |
| CD66b^High^ cells | 2.1 | 2.6 | 1.1 | 5.1 | 6.3 | 2.6 | 0.485 |
| CD66b^High^ / CD66b^Low^ ratio | 0.14 | 0.11 | 0.05 | 0.27 | 0.27 | 0.11 | 0.438 |
| CD163^+^ cells | 20.9 | 19.7 | 8.0 | 32.8 | 15.6 | 6.4 | 0.093 |
| CD56^Total^ cells | 50.6 | 21.4 | 8.7 | 57.8 | 21.0 | 8.6 | 0.485 |
| CD56^Low^ cells | 42.4 | 22.3 | 9.1 | 49.6 | 22.4 | 9.1 | 0.485 |
| CD56^High^ cells | 8.4 | 7.2 | 2.9 | 8.3 | 6.2 | 2.5 | 0.937 |
| CD56^High^ / CD56^Low^ ratio | 0.26 | 0.32 | 0.13 | 0.22 | 0.25 | 0.10 | 0.313 |
| CD66b^+^/CD56^+^ cells ratio | 0.4 | 0.3 | 0.1 | 0.4 | 0.4 | 0.2 | 0.937 |
| CD3^+^ cells | 11.4 | 10.2 | 4.2 | 7.5 | 7.6 | 3.1 | 0.699 |
| CD3^+^CD56^+^ cells | 10.3 | 10.4 | 4.2 | 15.7 | 10.2 | 4.2 | 0.288 |
| CD3^+^γδTCR^+^ cells | 1.6 | 2.2 | 0.9 | 3.4 | 2.5 | 1.0 | 0.169 |
