# Supplementary material for: Keratoconus patients exhibit a distinct ocular surface immune cell and inflammatory profile
Source: Sci Rep. 2021 Oct 22;11:20891. doi: 10.1038/s41598-021-99805-9 (PMC8536707; doi:10.1038/s41598-021-99805-9)
Supplement: Supplementary file 5 — Supplementary Table 2. [file 41598_2021_99805_MOESM5_ESM.docx]

**Supplementary Table 2:** **Tear fluid soluble factor levels in study subjects with** **forme fruste keratoconus in one eye and KC in the contralateral eye**

| **Analytes (pg/ml)** | **FFKC (n=6)** | | | **KC (n=6)** | | | **P value** |
| --- | --- | --- | --- | --- | --- | --- | --- |
|  | Mean | Stdev | SEM | Mean | Stdev | SEM |  |
| **Cytokines** |  |  |  |  |  |  |  |
| IL-1α | 14 | 10 | 4 | 25 | 42 | 17 | 0.699 |
| IL-1β | 6 | 8 | 3 | 6 | 7 | 3 | 0.818 |
| IL-2 | 124 | 143 | 58 | 120 | 135 | 55 | 0.935 |
| IL-6 | 22 | 32 | 13 | 21 | 23 | 9 | 0.699 |
| LIF | 972 | 753 | 308 | 1378 | 1761 | 719 | 0.699 |
| IL-9 | 0.0 | 0.0 | 0.0 | 12 | 30 | 12 | N.A |
| IL-10 | 0.6 | 0.9 | 0.4 | 1.3 | 1.5 | 0.6 | 0.180 |
| IL-12/IL23p40 | 922 | 1252 | 511 | 1462 | 1639 | 669 | 0.327 |
| IL-12p70 | 52 | 87 | 35 | 102 | 159 | 65 | 1.000 |
| IL-13 | 14 | 14 | 6 | 30 | 21 | 9 | 0.132 |
| IL-17A | 4 | 4 | 2 | 8 | 10 | 4 | 0.589 |
| IL-18 | 108 | 71 | 29 | 282 | 448 | 183 | 0.937 |
| IL-21 | 346 | 386 | 158 | 458 | 659 | 269 | 0.870 |
| TNFα | 1.8 | 1.6 | 0.7 | 8.9 | 14.9 | 6.1 | 0.387 |
| IFNα | 26 | 29 | 12 | 53 | 45 | 19 | 0.180 |
| IFNβ | 479 | 490 | 200 | 445 | 507 | 207 | 0.485 |
| IFNγ | 45 | 75 | 31 | 101 | 234 | 95 | 0.981 |
| **Chemokines** |  |  |  |  |  |  |  |
| MCP1/CCL2 | 98 | 57 | 23 | 158 | 117 | 48 | 0.394 |
| RANTES/CCL5 | 116 | 84 | 34 | 284 | 524 | 214 | 0.699 |
| MIG/CCL9 | 1298 | 2289 | 935 | 1204 | 2389 | 975 | 0.699 |
| Eotaxin/CCL11 | 19 | 23 | 10 | 31 | 34 | 14 | 0.671 |
| IL-8/CXCL8 | 649 | 744 | 304 | 535 | 275 | 112 | 0.589 |
| IP-10/CXCL10 (ng/ml) | 51 | 95 | 39 | 21 | 14 | 6 | 0.699 |
| ITAC/CXCL11 | 343 | 411 | 168 | 391 | 377 | 154 | 0.805 |
| Fractalkine/CX3CL1 | 2.8 | 2.8 | 1.2 | 3.2 | 3.5 | 1.4 | 0.859 |
| **Growth Factors** |  |  |  |  |  |  |  |
| TGFβ1 (ng/ml) | 7 | 9 | 4 | 10 | 12 | 5 | 0.606 |
| bFGF | 26 | 63 | 26 | 106 | 126 | 52 | 0.303 |
| HGF | 417 | 257 | 105 | 506 | 535 | 218 | 0.818 |
| EPO | 93 | 70 | 28 | 294 | 581 | 237 | 0.699 |
| PDGF-AA | 318 | 132 | 54 | 561 | 501 | 204 | 0.589 |
| PDGF-BB | 120 | 75 | 31 | 235 | 416 | 170 | 0.485 |
| VEGF | 801 | 746 | 304 | 1029 | 898 | 367 | 0.485 |
| **Soluble cell adhesion molecules and soluble receptors** | | | | | | | |
| sICAM1 (ng/ml) | 6.4 | 2.7 | 1.1 | 5.8 | 5.2 | 2.1 | 0.818 |
| sVCAM (ng/ml) | 1.9 | 1.6 | 0.6 | 1.6 | 1.2 | 0.5 | 0.937 |
| sL-selectin (ng/ml) | 7.8 | 11.7 | 4.8 | 2.9 | 2.6 | 1.1 | 0.394 |
| sP-selectin (ng/ml) | 0.1 | 0.1 | 0.0 | 0.1 | 0.1 | 0.1 | 0.948 |
| sTNFRI | 599 | 370 | 151 | 350 | 213 | 87 | 0.240 |
| sTNFRII | 21 | 13 | 5 | 23 | 17 | 7 | 0.818 |
| sIL-1R1 | 667 | 395 | 161 | 680 | 564 | 230 | 0.818 |
| **Enzymes** |  |  |  |  |  |  |  |
| MMP2 (ng/ml) | 2 | 1 | 0 | 8 | 16 | 6 | 0.937 |
| MMP9 (ng/ml) | 1050 | 1467 | 599 | 478 | 350 | 143 | 0.937 |
| TIMP1 (ng/ml) | 44 | 23 | 9 | 45 | 36 | 15 | 0.937 |
| MPO (ng/ml) | 130 | 241 | 98 | 56 | 67 | 27 | 0.818 |
| NGAL (ng/ml) | 413 | 388 | 158 | 819 | 1480 | 604 | 0.699 |
| Angiogenin (ng/ml) | 343 | 479 | 196 | 320 | 327 | 134 | 0.699 |
| **Other secreted factors** |  |  |  |  |  |  |  |
| Granzyme-B | 436 | 402 | 164 | 493 | 551 | 225 | 0.937 |
| Perforin | 283 | 575 | 235 | 479 | 946 | 386 | 0.937 |
| IgE | 505 | 775 | 316 | 239 | 225 | 92 | 0.937 |
| sFasL | 29 | 25 | 10 | 55 | 57 | 23 | 0.485 |
| β2 microglobulin (ng/ml) | 522 | 669 | 273 | 1761 | 3976 | 1623 | 0.589 |
