# Supplementary material for: Keratoconus patients exhibit a distinct ocular surface immune cell and inflammatory profile
Source: Sci Rep. 2021 Oct 22;11:20891. doi: 10.1038/s41598-021-99805-9 (PMC8536707; doi:10.1038/s41598-021-99805-9)
Supplement: Supplementary file 6 — Supplementary Table 3. [file 41598_2021_99805_MOESM6_ESM.docx]

**Supplementary Table 3:** **Ocular surface immune subset proportions in KC subjects with and without history of systemic allergy**

| **Ocular surface immune cell subset proportions** | **No history of systemic allergy (n=44)** | | | **History of systemic allergy (n=7)** | | | **P value** |
| --- | --- | --- | --- | --- | --- | --- | --- |
|  | Mean | Stdev | SEM | Mean | Stdev | SEM |  |
| CD45^+^ cells | 33.8 | 25.0 | 3.8 | 30.2 | 14.6 | 5.5 | 0.862 |
| CD66b^Total^ cells | 22.3 | 20.5 | 3.1 | 23.3 | 5.8 | 2.2 | 0.305 |
| CD66b^Low^ cells | 14.0 | 14.0 | 2.1 | 18.3 | 5.9 | 2.2 | 0.042 |
| CD66b^High^ cells | 8.6 | 15.4 | 2.3 | 5.4 | 4.9 | 1.9 | 0.691 |
| CD66b^High^ / CD66b^Low^ ratio | 1.2 | 2.7 | 0.4 | 0.3 | 0.3 | 0.1 | 0.880 |
| CD163^+^ cells | 29.9 | 19.2 | 2.9 | 33.3 | 17.0 | 6.4 | 0.556 |
| CD56^Total^ cells | 53.2 | 18.5 | 2.8 | 41.4 | 18.3 | 6.9 | 0.122 |
| CD56^Low^ cells | 41.8 | 17.2 | 2.6 | 29.0 | 13.4 | 5.1 | 0.052 |
| CD56^High^ cells | 11.8 | 8.2 | 1.2 | 12.7 | 14.0 | 5.3 | 0.779 |
| CD56^High^ / CD56^Low^ ratio | 0.3 | 0.2 | 0.0 | 0.5 | 0.8 | 0.3 | 0.862 |
| CD66b^+^/CD56^+^ cells ratio | 0.5 | 0.7 | 0.1 | 0.7 | 0.4 | 0.2 | 0.082 |
| CD3^+^ cells | 11.1 | 11.7 | 1.8 | 13.8 | 9.0 | 3.4 | 0.256 |
| CD3^+^CD56^+^ cells | 17.9 | 12.3 | 1.9 | 22.2 | 19.3 | 7.3 | 0.753 |
| CD3^+^γδTCR^+^ cells | 2.8 | 3.4 | 0.5 | 5.6 | 6.4 | 2.4 | 0.158 |
