# Supplementary material for: Keratoconus patients exhibit a distinct ocular surface immune cell and inflammatory profile
Source: Sci Rep. 2021 Oct 22;11:20891. doi: 10.1038/s41598-021-99805-9 (PMC8536707; doi:10.1038/s41598-021-99805-9)
Supplement: Supplementary file 7 — Supplementary Table 4. [file 41598_2021_99805_MOESM7_ESM.docx]

**Supplementary Table 4:** **Ocular surface immune subset proportions in KC subjects with and without mild ocular allergy**

| **Ocular surface immune cell subset proportions** | **No ocular allergy (n=15)** | | | **Mild ocular allergy (n=36)** | | | **P value** |
| --- | --- | --- | --- | --- | --- | --- | --- |
|  | Mean | Stdev | SEM | Mean | Stdev | SEM |  |
| CD45^+^ cells | 24.0 | 19.6 | 5.1 | 37.2 | 24.5 | 4.1 | 0.050 |
| CD66b^Total^ cells | 19.0 | 14.9 | 3.8 | 23.8 | 20.6 | 3.4 | 0.642 |
| CD66b^Low^ cells | 12.1 | 8.4 | 2.2 | 15.6 | 14.8 | 2.5 | 0.767 |
| CD66b^High^ cells | 6.9 | 9.2 | 2.4 | 8.7 | 16.2 | 2.7 | 0.756 |
| CD66b^High^ / CD66b^Low^ ratio | 0.7 | 1.2 | 0.3 | 1.3 | 2.9 | 0.5 | 0.657 |
| CD163^+^ cells | 34.5 | 22.8 | 5.9 | 28.6 | 17.0 | 2.8 | 0.451 |
| CD56^Total^ cells | 56.5 | 17.1 | 4.4 | 49.5 | 19.2 | 3.2 | 0.231 |
| CD56^Low^ cells | 43.6 | 17.8 | 4.6 | 38.6 | 17.0 | 2.8 | 0.506 |
| CD56^High^ cells | 13.4 | 9.7 | 2.5 | 11.3 | 8.8 | 1.5 | 0.467 |
| CD56^High^ / CD56^Low^ ratio | 0.4 | 0.3 | 0.1 | 0.3 | 0.4 | 0.1 | 0.493 |
| CD66b^+^/CD56^+^ cells ratio | 0.4 | 0.3 | 0.1 | 0.7 | 0.8 | 0.1 | 0.263 |
| CD3^+^ cells | 10.4 | 7.6 | 2.0 | 12.0 | 12.6 | 2.1 | 0.967 |
| CD3^+^CD56^+^ cells | 15.6 | 10.7 | 2.8 | 19.6 | 14.2 | 2.4 | 0.397 |
| CD3^+^γδTCR^+^ cells | 2.3 | 2.8 | 0.7 | 3.5 | 4.4 | 0.7 | 0.418 |
