# Supplementary material for: Keratoconus patients exhibit a distinct ocular surface immune cell and inflammatory profile
Source: Sci Rep. 2021 Oct 22;11:20891. doi: 10.1038/s41598-021-99805-9 (PMC8536707; doi:10.1038/s41598-021-99805-9)
Supplement: Supplementary file 8 — Supplementary Table 5. [file 41598_2021_99805_MOESM8_ESM.docx]

**Supplementary Table 5:** **Ocular surface immune subset proportions in KC subjects with and without history of eye rubbing**

| **Ocular surface immune cell subset proportions** | **No Eye rubbing (n=18)** | | | **Eye rubbing (n=33)** | | | **P value** |
| --- | --- | --- | --- | --- | --- | --- | --- |
|  | Mean | Stdev | SEM | Mean | Stdev | SEM |  |
| CD45^+^ cells | 20.0 | 14.9 | 3.5 | 40.6 | 24.6 | 4.3 | 0.004 |
| CD66b^Total^ cells | 21.9 | 17.0 | 4.0 | 22.7 | 20.4 | 3.5 | 0.953 |
| CD66b^Low^ cells | 14.4 | 11.2 | 2.7 | 14.7 | 14.4 | 2.5 | 0.853 |
| CD66b^High^ cells | 7.7 | 8.9 | 2.1 | 8.4 | 16.9 | 2.9 | 0.419 |
| CD66b^High^ / CD66b^Low^ ratio | 0.6 | 1.1 | 0.3 | 1.3 | 3.1 | 0.5 | 0.509 |
| CD163^+^ cells | 36.2 | 19.7 | 4.6 | 27.2 | 17.9 | 3.1 | 0.100 |
| CD56^Total^ cells | 57.7 | 17.1 | 4.0 | 48.2 | 19.0 | 3.3 | 0.076 |
| CD56^Low^ cells | 44.2 | 17.0 | 4.0 | 37.8 | 17.2 | 3.0 | 0.220 |
| CD56^High^ cells | 14.3 | 9.9 | 2.3 | 10.7 | 8.4 | 1.5 | 0.206 |
| CD56^High^ / CD56^Low^ ratio | 0.4 | 0.3 | 0.1 | 0.3 | 0.4 | 0.1 | 0.323 |
| CD66b^+^/CD56^+^ cells ratio | 0.4 | 0.3 | 0.1 | 0.7 | 0.8 | 0.1 | 0.525 |
| CD3^+^ cells | 9.2 | 7.3 | 1.7 | 12.8 | 12.9 | 2.2 | 0.466 |
| CD3^+^CD56^+^ cells | 16.7 | 11.0 | 2.6 | 19.4 | 14.5 | 2.5 | 0.622 |
| CD3^+^γδTCR^+^ cells | 1.9 | 2.6 | 0.6 | 3.9 | 4.5 | 0.8 | 0.106 |
