# Supplementary material for: Keratoconus patients exhibit a distinct ocular surface immune cell and inflammatory profile
Source: Sci Rep. 2021 Oct 22;11:20891. doi: 10.1038/s41598-021-99805-9 (PMC8536707; doi:10.1038/s41598-021-99805-9)
Supplement: Supplementary file 9 — Supplementary Table 6. [file 41598_2021_99805_MOESM9_ESM.docx]

**Supplementary Table 6: Tear fluid soluble factor levels in KC subjects with and without history of systemic allergy**

| **Analytes (pg/ml)** | **No history of systemic allergy (n=35)** | | | **History of systemic allergy (n=6)** | | | **P value** |
| --- | --- | --- | --- | --- | --- | --- | --- |
|  | Mean | Stdev | SEM | Mean | Stdev | SEM |  |
| **Cytokines** |  |  |  |  |  |  |  |
| IL-1α | 19.4 | 24.5 | 4.1 | 32.4 | 21.7 | 8.9 | 0.028 |
| IL-1β | 3.7 | 4.8 | 0.8 | 20.9 | 37.0 | 15.1 | 0.698 |
| IL-2 | 99.4 | 121.8 | 20.6 | 129.6 | 94.6 | 38.6 | 0.352 |
| IL-6 | 19.4 | 26.5 | 4.5 | 91.8 | 177.8 | 72.6 | 0.706 |
| LIF | 782.1 | 866.2 | 146.4 | 1085.8 | 1301.3 | 531.2 | 0.574 |
| IL-9 | 25.1 | 81.8 | 13.8 | 48.6 | 75.7 | 30.9 | <0.0001 |
| IL-10 | 2.3 | 3.2 | 0.5 | 5.2 | 3.3 | 1.4 | 0.016 |
| IL-12/IL23p40 | 2116.4 | 3069.9 | 518.9 | 3138.4 | 1558.6 | 636.3 | 0.091 |
| IL-12p70 | 192.8 | 355.7 | 60.1 | 625.6 | 637.2 | 260.1 | 0.093 |
| IL-13 | 33.9 | 45.0 | 7.6 | 54.8 | 22.3 | 9.1 | 0.029 |
| IL-17A | 7.2 | 8.2 | 1.4 | 13.4 | 10.1 | 4.1 | 0.100 |
| IL-18 | 97.3 | 196.8 | 33.3 | 512.6 | 770.6 | 314.6 | 0.573 |
| IL-21 | 808.6 | 1928.5 | 326.0 | 809.2 | 952.0 | 388.6 | 0.508 |
| TNFα | 7.0 | 11.6 | 2.0 | 21.6 | 18.1 | 7.4 | 0.007 |
| IFNα | 45.5 | 43.3 | 7.3 | 69.2 | 46.1 | 18.8 | 0.171 |
| IFNβ | 302.8 | 334.6 | 56.5 | 427.6 | 518.1 | 211.5 | 0.963 |
| IFNγ | 30.1 | 101.2 | 17.1 | 64.3 | 105.5 | 43.1 | 0.958 |
| **Chemokines** |  |  |  |  |  |  |  |
| MCP1/CCL2 | 160.5 | 141.7 | 24.7 | 278.4 | 117.2 | 47.9 | 0.063 |
| RANTES/CCL5 | 112.4 | 227.0 | 38.4 | 173.9 | 260.5 | 106.4 | 0.925 |
| Eotaxin/CCL11 | 542.3 | 471.8 | 79.8 | 3156.8 | 6191.8 | 2527.8 | 0.706 |
| IL-8/CXCL8 | 8422.4 | 44004.1 | 7438.0 | 1974.4 | 3462.1 | 1413.4 | 0.209 |
| MIG/CXCL9 | 37.6 | 40.0 | 6.8 | 55.6 | 49.7 | 20.3 | 0.496 |
| IP-10/CXCL10 (ng/ml) | 180.6 | 725.1 | 122.6 | 58138.7 | 142250.5 | 58073.5 | 0.171 |
| ITAC/CXCL11 | 565.7 | 584.4 | 101.7 | 875.8 | 711.0 | 290.3 | 0.358 |
| Fractalkine/CX3CL1 | 216.7 | 1167.3 | 197.3 | 6.7 | 3.3 | 1.5 | 0.092 |
| **Growth Factors** |  |  |  |  |  |  |  |
| TGFβ1 (ng/ml) | 15.0 | 22.0 | 3.7 | 32.9 | 31.1 | 12.7 | 0.252 |
| bFGF | 150.4 | 232.0 | 39.2 | 245.1 | 216.1 | 88.2 | 0.227 |
| HGF | 339.3 | 329.1 | 55.6 | 507.8 | 647.0 | 264.1 | 0.580 |
| EPO | 127.7 | 251.5 | 42.5 | 219.6 | 300.3 | 122.6 | 0.868 |
| PDGF-AA | 294.6 | 267.8 | 45.3 | 476.6 | 465.6 | 190.1 | 0.356 |
| PDGF-BB | 115.2 | 178.7 | 30.2 | 227.7 | 312.1 | 127.4 | 0.758 |
| VEGF | 1198.1 | 1061.6 | 179.4 | 2178.1 | 1819.1 | 742.6 | 0.171 |
| **Soluble cell adhesion molecules and soluble receptors** | | | | | | | |
| sICAM1 (ng/ml) | 4.4 | 3.5 | 0.6 | 14.9 | 22.1 | 9.0 | 0.128 |
| sVCAM (ng/ml) | 2.0 | 2.0 | 0.3 | 9.8 | 13.0 | 5.3 | 0.020 |
| sL-selectin (ng/ml) | 7.2 | 20.7 | 3.5 | 24.3 | 41.5 | 16.9 | 0.180 |
| sP-selectin (ng/ml) | 0.4 | 0.5 | 0.1 | 0.8 | 0.8 | 0.3 | 0.077 |
| sTNFRI | 273.1 | 263.6 | 44.6 | 1478.7 | 2257.8 | 921.8 | 0.196 |
| sTNFRII | 26.1 | 28.1 | 4.7 | 184.6 | 309.2 | 126.2 | 0.017 |
| sIL-1R1 | 460.1 | 379.4 | 64.1 | 1138.8 | 1398.8 | 571.1 | 0.416 |
| **Enzymes** |  |  |  |  |  |  |  |
| MMP2 (ng/ml) | 3.3 | 6.7 | 1.1 | 7.7 | 11.1 | 4.5 | 0.928 |
| MMP9 (ng/ml) | 487.6 | 792.7 | 134.0 | 7714.3 | 14782.4 | 6034.9 | 0.577 |
| TIMP1 (ng/ml) | 46.8 | 34.6 | 5.8 | 73.0 | 104.3 | 42.6 | 0.706 |
| MPO (ng/ml) | 61.3 | 121.2 | 20.5 | 734.9 | 1387.7 | 566.5 | 0.378 |
| NGAL (ng/ml) | 405.6 | 727.6 | 123.0 | 739.7 | 1010.9 | 412.7 | 0.713 |
| Angiogenin (ng/ml) | 473.1 | 692.5 | 117.1 | 2097.8 | 2812.8 | 1148.3 | 0.080 |
| **Other secreted factors** |  |  |  |  |  |  |  |
| Granzyme-B | 354.4 | 481.2 | 81.3 | 372.7 | 486.7 | 198.7 | 0.268 |
| Perforin | 251.7 | 542.5 | 91.7 | 56.8 | 66.6 | 27.2 | 0.310 |
| IgE | 186.5 | 379.7 | 65.1 | 2098.1 | 3697.4 | 1509.4 | 0.037 |
| sFasL | 24.2 | 30.1 | 5.1 | 42.1 | 51.4 | 21.0 | 0.323 |
| β2 microglobulin (ng/ml) | 481.6 | 1673.8 | 282.9 | 1463.9 | 2463.8 | 1005.9 | 0.986 |
