# Supplementary material for: Keratoconus patients exhibit a distinct ocular surface immune cell and inflammatory profile
Source: Sci Rep. 2021 Oct 22;11:20891. doi: 10.1038/s41598-021-99805-9 (PMC8536707; doi:10.1038/s41598-021-99805-9)
Supplement: Supplementary file 10 — Supplementary Table 7. [file 41598_2021_99805_MOESM10_ESM.docx]

**Supplementary Table 7: Tear fluid soluble factor levels in KC subjects with and without mild ocular allergy**

| **Analytes (pg/ml)** | **No ocular allergy (n13)** | | | **Mild ocular allergy (n=28)** | | | **P value** |
| --- | --- | --- | --- | --- | --- | --- | --- |
|  | Mean | Stdev | SEM | Mean | Stdev | SEM |  |
| **Cytokines** |  |  |  |  |  |  |  |
| IL-1α | 17.3 | 29.2 | 8.1 | 23.1 | 22.0 | 4.1 | 0.052 |
| IL-1β | 6.0 | 7.1 | 2.0 | 6.5 | 18.1 | 3.5 | 0.407 |
| IL-2 | 51.2 | 102.4 | 28.4 | 128.2 | 117.9 | 22.3 | 0.007 |
| IL-6 | 26.1 | 28.7 | 8.0 | 31.7 | 85.8 | 16.2 | 0.216 |
| LIF | 985.3 | 1301.5 | 361.0 | 752.8 | 710.8 | 134.3 | 0.779 |
| IL-9 | 48.2 | 123.3 | 34.2 | 19.4 | 50.6 | 9.6 | 0.736 |
| IL-10 | 0.9 | 0.8 | 0.2 | 3.5 | 3.7 | 0.7 | 0.054 |
| IL-12/IL23p40 | 2152.4 | 4682.3 | 1298.6 | 2318.6 | 1662.2 | 314.1 | 0.041 |
| IL-12p70 | 26.8 | 51.6 | 14.3 | 362.6 | 481.4 | 91.0 | 0.002 |
| IL-13 | 35.0 | 68.2 | 18.9 | 37.9 | 25.5 | 4.8 | 0.038 |
| IL-17A | 4.9 | 6.7 | 1.9 | 9.6 | 9.2 | 1.7 | 0.046 |
| IL-18 | 181.8 | 310.3 | 86.1 | 147.0 | 385.0 | 72.8 | 0.327 |
| IL-21 | 1027.5 | 3043.2 | 844.0 | 707.1 | 837.4 | 158.3 | 0.122 |
| TNFα | 4.8 | 10.4 | 2.9 | 11.2 | 14.5 | 2.7 | 0.115 |
| IFNα | 25.4 | 30.9 | 8.6 | 59.9 | 45.2 | 8.5 | 0.021 |
| IFNβ | 250.1 | 343.7 | 95.3 | 354.1 | 370.9 | 70.1 | 0.179 |
| IFNγ | 65.7 | 162.7 | 45.1 | 20.9 | 52.5 | 9.9 | 0.255 |
| **Chemokines** |  |  |  |  |  |  |  |
| MCP1/CCL2 | 138.8 | 114.7 | 34.6 | 194.3 | 152.2 | 28.8 | 0.414 |
| RANTES/CCL5 | 191.3 | 356.1 | 98.8 | 89.0 | 135.6 | 25.6 | 0.218 |
| Eotaxin/CCL11 | 656.8 | 488.6 | 135.5 | 1049.3 | 2918.9 | 551.6 | 0.151 |
| IL-8/CXCL8 | 21847.8 | 71936.1 | 19951.5 | 807.5 | 1734.0 | 327.7 | 0.128 |
| MIG/CXCL9 | 16.1 | 20.9 | 5.8 | 51.4 | 43.9 | 8.3 | 0.006 |
| IP-10/CXCL10 (ng/ml) | 64.9 | 108.4 | 30.1 | 12653.9 | 65825.8 | 12439.9 | 0.430 |
| ITAC/CXCL11 | 208.0 | 266.5 | 80.3 | 772.6 | 630.0 | 119.1 | 0.004 |
| Fractalkine/CX3CL1 | 576.3 | 1907.3 | 529.0 | 4.7 | 3.1 | 0.6 | 0.105 |
| **Growth Factors** |  |  |  |  |  |  |  |
| TGFβ1 (ng/ml) | 3.2 | 5.3 | 1.5 | 24.3 | 26.3 | 5.0 | 0.001 |
| bFGF | 132.9 | 310.3 | 86.1 | 178.9 | 186.1 | 35.2 | 0.137 |
| HGF | 463.3 | 490.5 | 136.1 | 317.8 | 324.6 | 61.3 | 0.400 |
| EPO | 226.6 | 395.9 | 109.8 | 101.5 | 152.1 | 28.7 | 0.044 |
| PDGF-AA | 414.9 | 362.3 | 100.5 | 277.8 | 268.8 | 50.8 | 0.107 |
| PDGF-BB | 154.4 | 285.6 | 79.2 | 121.2 | 155.0 | 29.3 | 0.812 |
| VEGF | 830.8 | 1024.6 | 284.2 | 1578.6 | 1250.5 | 236.3 | 0.021 |
| **Soluble cell adhesion molecules and soluble receptors** | | | | | | | |
| sICAM1 (ng/ml) | 5.4 | 4.0 | 1.1 | 6.2 | 10.9 | 2.1 | 0.552 |
| sVCAM (ng/ml) | 2.3 | 2.3 | 0.6 | 3.6 | 6.7 | 1.3 | 0.900 |
| sL-selectin (ng/ml) | 16.1 | 32.6 | 9.1 | 6.7 | 20.2 | 3.8 | 0.080 |
| sP-selectin (ng/ml) | 0.4 | 0.7 | 0.2 | 0.4 | 0.5 | 0.1 | 0.223 |
| sTNFRI | 329.8 | 319.1 | 88.5 | 505.1 | 1118.9 | 211.4 | 0.552 |
| sTNFRII | 30.8 | 34.2 | 9.5 | 57.9 | 150.7 | 28.5 | 0.922 |
| sIL-1R1 | 602.0 | 514.2 | 142.6 | 539.6 | 715.7 | 135.3 | 0.430 |
| **Enzymes** |  |  |  |  |  |  |  |
| MMP2 (ng/ml) | 4.7 | 10.7 | 3.0 | 3.6 | 5.6 | 1.1 | 0.750 |
| MMP9 (ng/ml) | 712.0 | 1063.9 | 295.1 | 1932.0 | 7083.2 | 1338.6 | 0.324 |
| TIMP1 (ng/ml) | 59.1 | 38.4 | 10.7 | 46.7 | 54.3 | 10.3 | 0.135 |
| MPO (ng/ml) | 111.4 | 168.3 | 46.7 | 182.3 | 668.5 | 126.3 | 0.034 |
| NGAL (ng/ml) | 777.8 | 1113.4 | 308.8 | 304.4 | 502.4 | 94.9 | 0.031 |
| Angiogenin (ng/ml) | 171.7 | 175.4 | 48.7 | 961.1 | 1533.4 | 289.8 | 0.013 |
| **Other secreted factors** |  |  |  |  |  |  |  |
| Granzyme-B | 464.0 | 706.5 | 195.9 | 307.4 | 325.1 | 61.4 | 0.878 |
| Perforin | 64.3 | 91.5 | 25.4 | 296.9 | 597.5 | 112.9 | 0.348 |
| IgE | 362.0 | 566.9 | 157.2 | 526.8 | 1836.1 | 353.4 | 0.820 |
| sFasL | 32.4 | 46.5 | 12.9 | 24.2 | 26.7 | 5.0 | 0.623 |
| β2 microglobulin (ng/ml) | 998.1 | 2704.1 | 750.0 | 452.3 | 1219.5 | 230.5 | 0.102 |
