# Supplementary material for: Keratoconus patients exhibit a distinct ocular surface immune cell and inflammatory profile
Source: Sci Rep. 2021 Oct 22;11:20891. doi: 10.1038/s41598-021-99805-9 (PMC8536707; doi:10.1038/s41598-021-99805-9)
Supplement: Supplementary file 11 — Supplementary Table 8. [file 41598_2021_99805_MOESM11_ESM.docx]

**Supplementary Table 8: Tear fluid soluble factor levels in KC subjects with and without history of eye rubbing**

| **Analytes (pg/ml)** | **No Eye rubbing (n=14)** | | | **Eye rubbing (n=27)** | | | **P value** |
| --- | --- | --- | --- | --- | --- | --- | --- |
|  | Mean | Stdev | SEM | Mean | Stdev | SEM |  |
| **Cytokines** |  |  |  |  |  |  |  |
| IL-1α | 17 | 28 | 8 | 23 | 22 | 4 | 0.077 |
| IL-1β | 6 | 7 | 2 | 6 | 18 | 4 | 0.146 |
| IL-2 | 25 | 64 | 17 | 145 | 119 | 23 | <0.0001 |
| IL-6 | 29 | 27 | 7 | 31 | 88 | 17 | 0.014 |
| LIF | 962 | 1253 | 335 | 756 | 724 | 139 | 0.978 |
| IL-9 | 44.8 | 119.1 | 31.8 | 20 | 51 | 10 | 0.8 |
| IL-10 | 1.1 | 0.9 | 0.3 | 3.5 | 3.8 | 0.7 | 0.149 |
| IL-12/IL23p40 | 2086 | 4506 | 1204 | 2359 | 1679 | 323 | 0.032 |
| IL-12p70 | 28 | 49 | 13 | 375 | 486 | 94 | 0.003 |
| IL-13 | 34 | 66 | 18 | 38 | 26 | 5 | 0.042 |
| IL-17A | 5 | 6 | 2 | 10 | 9 | 2 | 0.086 |
| IL-18 | 168 | 302 | 81 | 153 | 391 | 75 | 0.441 |
| IL-21 | 975 | 2931 | 783 | 723 | 849 | 163 | 0.129 |
| TNFα | 4.9 | 9.9 | 2.7 | 11.3 | 14.7 | 2.8 | 0.265 |
| IFNα | 26 | 30 | 8 | 61 | 46 | 9 | 0.019 |
| IFNβ | 249 | 330 | 88 | 358 | 377 | 73 | 0.284 |
| IFNγ | 62 | 157 | 42 | 21 | 53 | 10 | 0.289 |
| **Chemokines** |  |  |  |  |  |  |  |
| MCP1/CCL2 | 183 | 130 | 38 | 177 | 151 | 29 | 0.753 |
| RANTES/CCL5 | 174 | 346 | 92 | 94 | 138 | 27 | 0.441 |
| Eotaxin/CCL11 | 15 | 20 | 5 | 53 | 44 | 8 | 0.003 |
| IL-8/CXCL8 | 797 | 604 | 161 | 991 | 2969 | 571 | 0.034 |
| MIG/CXCL9 | 20580 | 69273 | 18514 | 686 | 1716 | 330 | 0.020 |
| IP-10/CXCL10 (ng/ml) | 62 | 105 | 28 | 13122 | 67032 | 12900 | 0.505 |
| ITAC/CXCL11 | 229 | 255 | 74 | 784 | 641 | 123 | 0.008 |
| Fractalkine/CX3CL1 | 535.4 | 1838.9 | 491.5 | 4.7 | 3.1 | 0.6 | 0.108 |
| **Growth Factors** |  |  |  |  |  |  |  |
| TGFβ1 (ng/ml) | 3 | 5 | 1 | 25 | 27 | 5 | 0.001 |
| bFGF | 123 | 300 | 80 | 186 | 186 | 36 | 0.076 |
| HGF | 445 | 476 | 127 | 322 | 330 | 64 | 0.458 |
| EPO | 220 | 381 | 102 | 100 | 155 | 30 | 0.024 |
| PDGF-AA | 381 | 366 | 98 | 291 | 268 | 52 | 0.372 |
| PDGF-BB | 161 | 276 | 74 | 116 | 155 | 30 | 1.000 |
| VEGF | 791 | 991 | 265 | 1627 | 1249 | 240 | 0.006 |
| **Soluble cell adhesion molecules and soluble receptors** | | | | | | | |
| sICAM1 (ng/ml) | 6.3 | 4.2 | 1.1 | 5.8 | 11.1 | 2.1 | 0.115 |
| sVCAM (ng/ml) | 2.1 | 2.3 | 0.6 | 3.7 | 6.8 | 1.3 | 0.536 |
| sL-selectin (ng/ml) | 14.4 | 31.8 | 8.5 | 7.2 | 20.6 | 4.0 | 0.466 |
| sP-selectin (ng/ml) | 0.3 | 0.7 | 0.2 | 0.5 | 0.5 | 0.1 | 0.140 |
| sTNFRI | 280 | 316 | 84 | 537 | 1133 | 218 | 0.596 |
| sTNFRII | 27 | 34 | 9 | 61 | 153 | 29 | 0.573 |
| sIL-1R1 | 588 | 495 | 132 | 544 | 729 | 140 | 0.391 |
| **Enzymes** |  |  |  |  |  |  |  |
| MMP2 (ng/ml) | 5 | 10 | 3 | 3 | 6 | 1 | 0.559 |
| MMP9 (ng/ml) | 585 | 1018 | 272 | 2043 | 7201 | 1386 | 0.694 |
| TIMP1 (ng/ml) | 65 | 40 | 11 | 43 | 53 | 10 | 0.030 |
| MPO (ng/ml) | 88 | 162 | 43 | 197 | 679 | 131 | 0.134 |
| NGAL (ng/ml) | 739 | 1079 | 288 | 307 | 512 | 98 | 0.030 |
| Angiogenin (ng/ml) | 147 | 154 | 41 | 1003 | 1548 | 298 | 0.002 |
| **Other secreted factors** |  |  |  |  |  |  |  |
| Granzyme-B | 450 | 681 | 182 | 309 | 331 | 64 | 0.710 |
| Perforin | 64 | 88 | 23 | 306 | 607 | 117 | 0.670 |
| IgE | 338 | 552 | 148 | 546 | 1870 | 367 | 0.967 |
| sFasL | 31 | 45 | 12 | 25 | 27 | 5 | 0.572 |
| β2 microglobulin (ng/ml) | 930 | 2610 | 698 | 467 | 1240 | 239 | 0.108 |
